# Hybrid AC/DC microgrid test system simulation: grid-connected mode

**DOI:** 10.1016/j.heliyon.2019.e02862

**Published:** 2019-12-07

**Authors:** Leony Ortiz, Rogelio Orizondo, Alexander Águila, Jorge W. González, Gabriel J. López, Idi Isaac

**Affiliations:** a Carrera de Ingeniería Eléctrica, Grupo de Investigación GIREI, Universidad Politécnica Salesiana, Quito, Ecuador; b Carrera de Ingeniería Eléctrica, Grupo de Investigación TyD, Universidad Pontificia Bolivariana, Medellín, Colombia

**Keywords:** Electrical engineering, System diagnostics, Power system operation, Power converter, Smart grid technology, Distributed resources, Microgrid benchmark, Hybrid energy systems, Power flow

## Abstract

In this paper, a Microgrid (MG) test model based on the 14-busbar IEEE distribution system is proposed. This model can constitute an important research tool for the analysis of electrical grids in its transition to Smart Grids (SG). The benchmark is used as a base case for power flow analysis and quality variables related with SG and holds distributed resources. The proposed MG consists of DC and AC buses with different types of loads and distributed generation at two voltage levels. A complete model of this MG has been simulated using the MATLAB/Simulink environmental simulation platform. The proposed electrical system will provide a base case for other studies such as: reactive power compensation, stability and inertia analysis, reliability, demand response studies, hierarchical control, fault tolerant control, optimization and energy storage strategies.

## Teaser

Electrical engineering; System diagnostics; Power system operation; Power converter; Smart grid technology; Microgridsmart grids; Distributed resources; Microgrid benchmark; Hybrid energy systems; Power flow.

## 1 Introduction

Renewable energy systems (RES) propose a new technology that is cleaner and capable of supplying the growing electricity demands of interconnected and isolated communities. In recent years, MGs have become a great attraction for the scientific community as well as a promising solution for future traditional energy systems. MGs are seen as a possible technology for the integration of variable renewable energy systems in the traditional grid.

Currently, with the evolution of new digital technologies, such as micro-processed systems and advances in power electronics, many applications have been implemented in SG, specifically in the development of controllers and electronic energy converters. In recent decades, researchers have made significant contributions which have had a high impact in these areas, mainly aimed at data acquisition, automation, and control of MGs [1, 2, 3]. MGs not only integrate the distributed generation to the Main Grid in a reliable and clean fashion, but also provide high reliability in its capacity to operate in the face of natural phenomena and active Distribution Grids, which in turn results in less energy losses in transmission and distribution and less construction and investment time [3, 4, 5, 6]. Research developed in [7, 8, 9, 10, 11, 12, 13, 14, 15] show actual implemented MGs. Some of the examples can be seen in CERTS in the US, NEDO in Japan, and a vast majority of MGs in Europe.

A MG can be defined as a low-voltage distribution power system to which small modular generations systems, such as renewable energy sources, other distributed generators, as well as intermediate storage units are connected and can fulfill the load demand. This particular power system can be treated by the utility grid as a controllable load or generator [16]. Although MG configurations can be exclusively DC, AC, or a hybrid of the two technologies, some investigations are particularly focused on AC MG. This is due to its ability to operate together with the Main Grid [4, 17, 18, 19, 20, 21, 22]. It is known that each have several advantages which in turn leaves a HMG benchmark a turning point in this investigation [18, 23].

All MG technologies must face the dynamics and steady state characteristics of the distribution generators (DG), the unbalance and nonlinearity of loads and the proper dynamics of energy storage systems (ESS) [17]. HMGs must also face the problem of an accidental or a programmed disconnection from the Main Grid. An HMG benchmark must be subjected to two typical scenarios, as in any other power system Distribution Grid: minimum and maximum demand situations. HMGs must manage abnormal operations of its electrical infrastructure. A set of three-level control structures is a well-known strategy to control MG parameters [17, 30]. Specialized literature has reported implemented and experimental MGs in Europe, Africa, Asia and America [31, 32, 33, 34, 35, 36]. In fact, there is also a discussion about its acceptance [37]. Power electronics are included in MG configurations due to the nature of most renewable generation technologies. It is necessary to control the injected power delivered to the MG [17, 23]. If appropriate closed-loop control strategies are implemented, its power quality problems may be overcome [24, 25, 26]. The performance of the MG configurations and different technologies may be improved by the use of parallel inverters [27].

In this study, a detailed model of a Hybrid Microgrid (HMG) benchmark has been simulated. This model is based on the original IEEE-14-distribution-bus model. The proposed benchmark does not offer any wind energy resource since the effort is focused on the ability of the MG to operate one of two renewables energies. This is usually the case, as one of the two energy resources is more available than the other. The main objective of providing this benchmark is to set up a complete and detailed model for further studies: reliability and resilience, optimization, fault diagnosis, system identification, and fault tolerant control. There are many control techniques for the operation of controlled rectifiers or inverters, as interfaces to renewable energies. In this paper, some converters have been settled to operate in an open-loop control strategy and others operate in a closed-loop control strategy. Additionally, these rectifiers or inverters use pulse width modulation techniques of different carrier frequencies [17, 28, 29].

The MG is provided with typical balanced and unbalanced loads, linear and non-linear loads, energy storage systems, as well as distribution transformers and line impedances. This paper also offers a thorough description of the model with all the necessary data to tackle the aforementioned studies. Both demand scenarios are analyzed and a discussion about raised and potential problems and their possible solutions are also discussed. This study furnishes all power flow results for both scenarios. Next, the results show the conflicts that these variables may present with the bidirectionality of power flows and with the insertion of predominantly solar photovoltaic distributed generation. Lastly, the present paper is a starting point for the analysis of many current issues that will be described in future research.

The paper is distributed as follows: Section 2 explains the main electrical characteristics of MGs and its architecture. Section 3 describes every component of the proposed MG benchmark. In its subsections, details are given for the photovoltaic subsystems (3.1) and the battery energy storage subsystems (3.2). There is a complete description of the AC/DC voltage source converters (VSC) outside the DC bus (3.3 and 3.8) as well as their coupling transformers (3.4). Subsections 3.5, 3.6 and 3.7 are devoted to outline the electric characteristics of primary and secondary lines in addition to both linear and non-linear loads. Subsection 3.8 sketches the DC bus and all its parts, including their dual DC to AC converters and both a boost converter and a buck-boost converter. Section 4 shows load-flow solutions obtained from the simulation in MATLAB/Simulink. The results have been tabulated for the two opposite situations: a maximum demand scenario and a minimum demand scenario. Section 5 analyses both scenarios and future research areas are shown in Section 6. Finally, the conclusions can be found in Section 7.

## 2 Electric microgrids

MGs, even on a smaller scale, represent one of the most interesting solutions for researchers. MGs improve power flow in Distribution Grids and reduce power losses in transmission lines through DG interconnection, renewable energy sources (RES), battery energy storage systems (BESS) and loads [4, 12]. In the literature, several reviews direct to different control strategies [38, 39, 40, 41, 42, 43, 44], such as: test beds [8, 12], optimization techniques, and available software tools [45, 46], among others. Although MGs are considered on a small scale, their technical complexity on modelling and simulation is higher compared to the conventional energy system. Therefore, models that allow dynamic analysis are a key point to ensure that future MGs work in a stable way.

### 2.1 Microgrid architectures

**Fig. 1:**
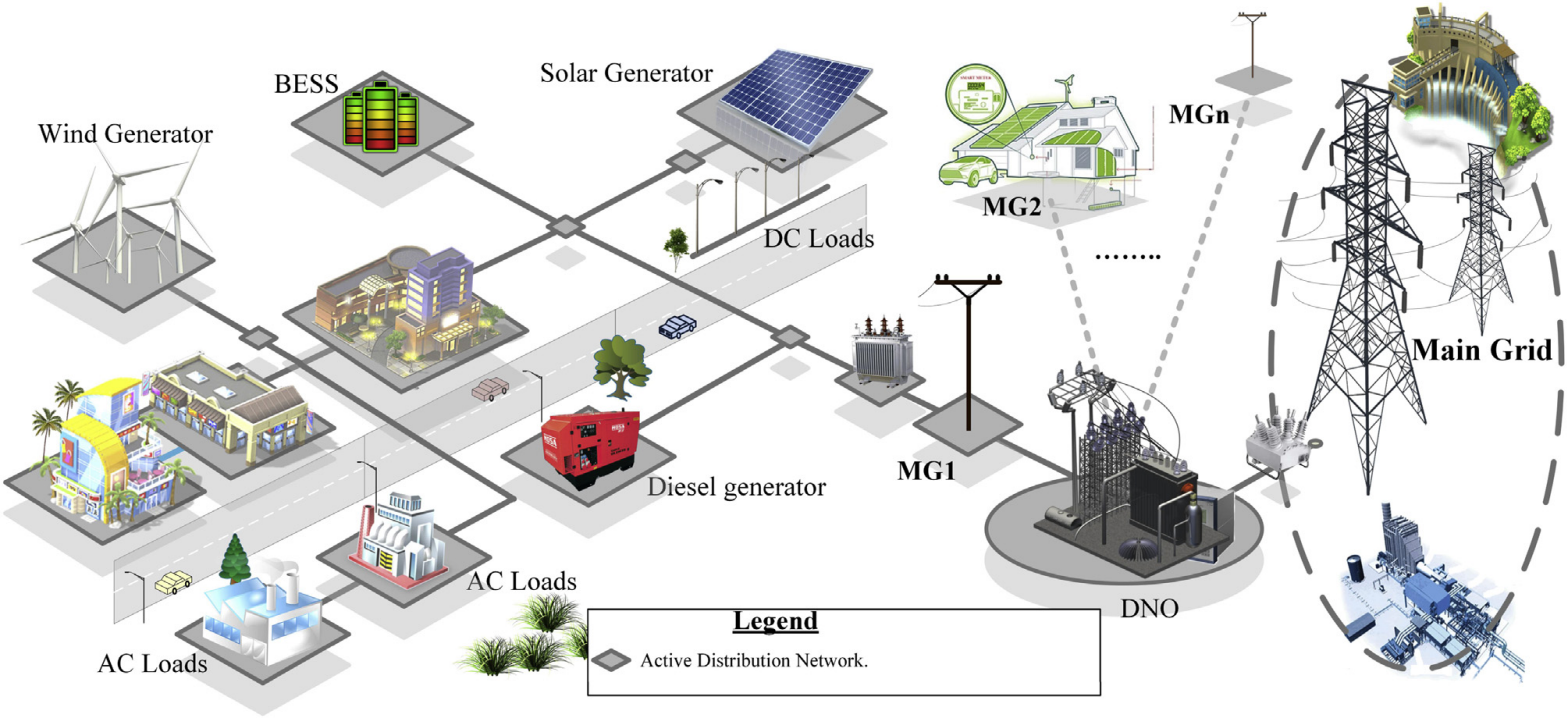
Electrical MG.

As shown in Fig. 1, MGs are constituted by a series of systems and subsystems such as: distributed generation, energy storage and different types of loads. The MGs can operate in parallel to the Main Grid, with no power exchange; in isolated mode, with autonomous power supplies; and in an interconnected mode, where it assumes the Main Grid set points [1, 3, 4, 5, 11, 20, 38, 41, 47, 48, 49]***.***

On the other hand, MGs can also be classified by attending to the following types:

• Types of voltage: DC; AC and hybrid.
• Distribution configuration: single-phase; three-phase; three-phase + neutral.
• Voltages: low (LV) and medium (MV).
• Structures: radial and ring.

**Fig. 2:**
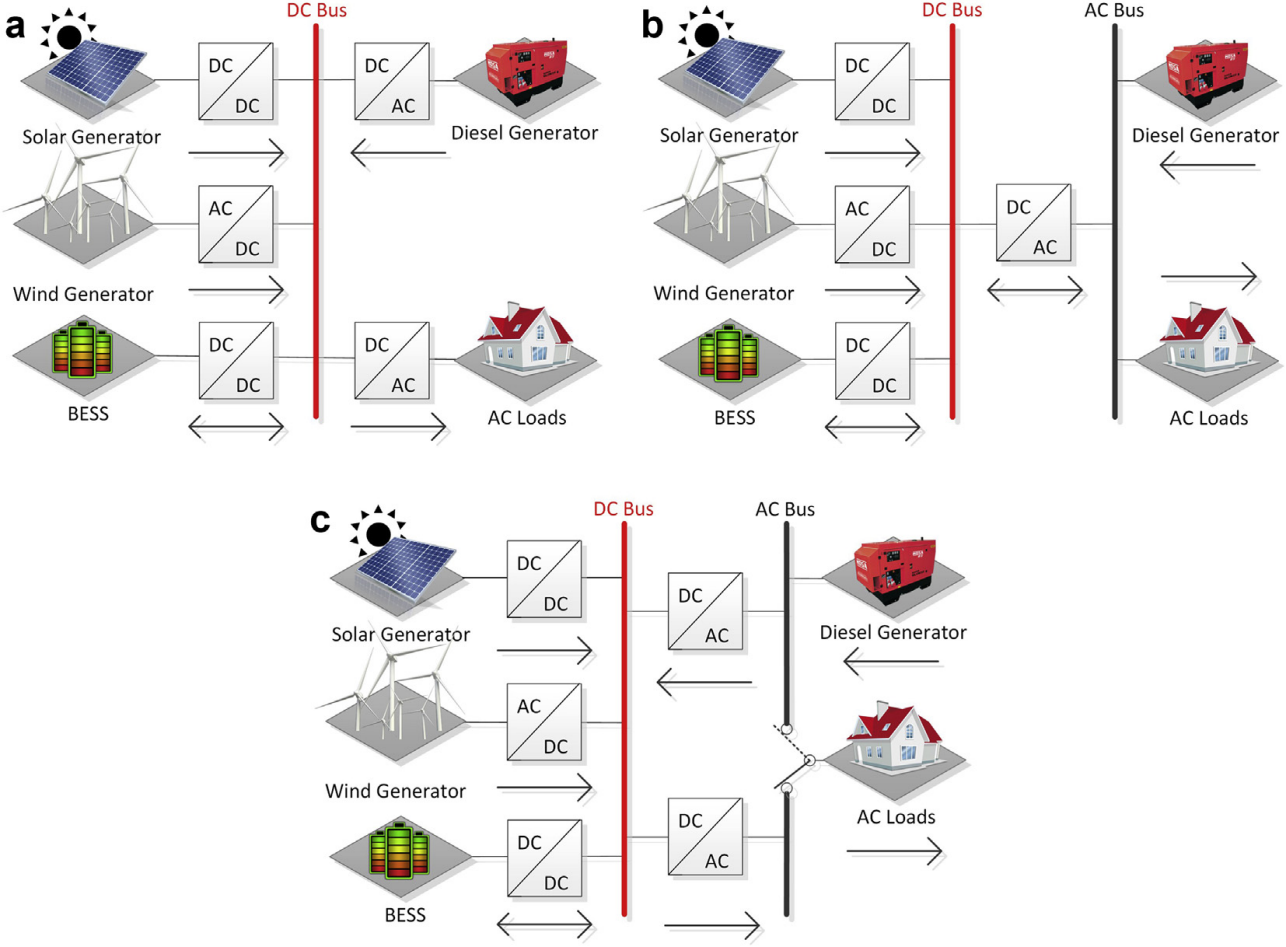
AC/DC MG configurations.

In the literature, three definitions are listed: Microgrid, Nanogrids, and Picogrids. As described in [7] and shown in Fig. 2, AC/DC MGs are usually implemented in series, switched, parallel or in combination. For the MG series configuration (Fig. 2a), there is a DC bus where all the generation systems and loads are connected through their respective converters.

The parallel configuration (Fig. 2b), has an AC bus where the generation systems and the loads are directly connected. The DC devices are connected through their own inverters, or through a DC bus coupled to the AC bus through an inverter or bidirectional VSC. Finally, in the switched configuration (Fig. 2c), the load can be supplied by a DG source or by the Distribution Grid (never in both cases at the same time), and both the DC MG and the AC MG are connected by two inverters [7, 47].

The configuration for MGs are mostly used in AC. However, DC MGs have gained much interest in recent times due to the advantages offered, such as a lack of reactive power and harmonics. Also, there is no need to synchronize the DC generation, for it presents few power losses, and there are no changes in the DC bus after a blackout. However, despite of all these advantages, disadvantages are present as well, and mainly, is the absence of crossing points by zero and the fact that the protection systems are more complex for high voltage levels [7].

**Fig. 3:**
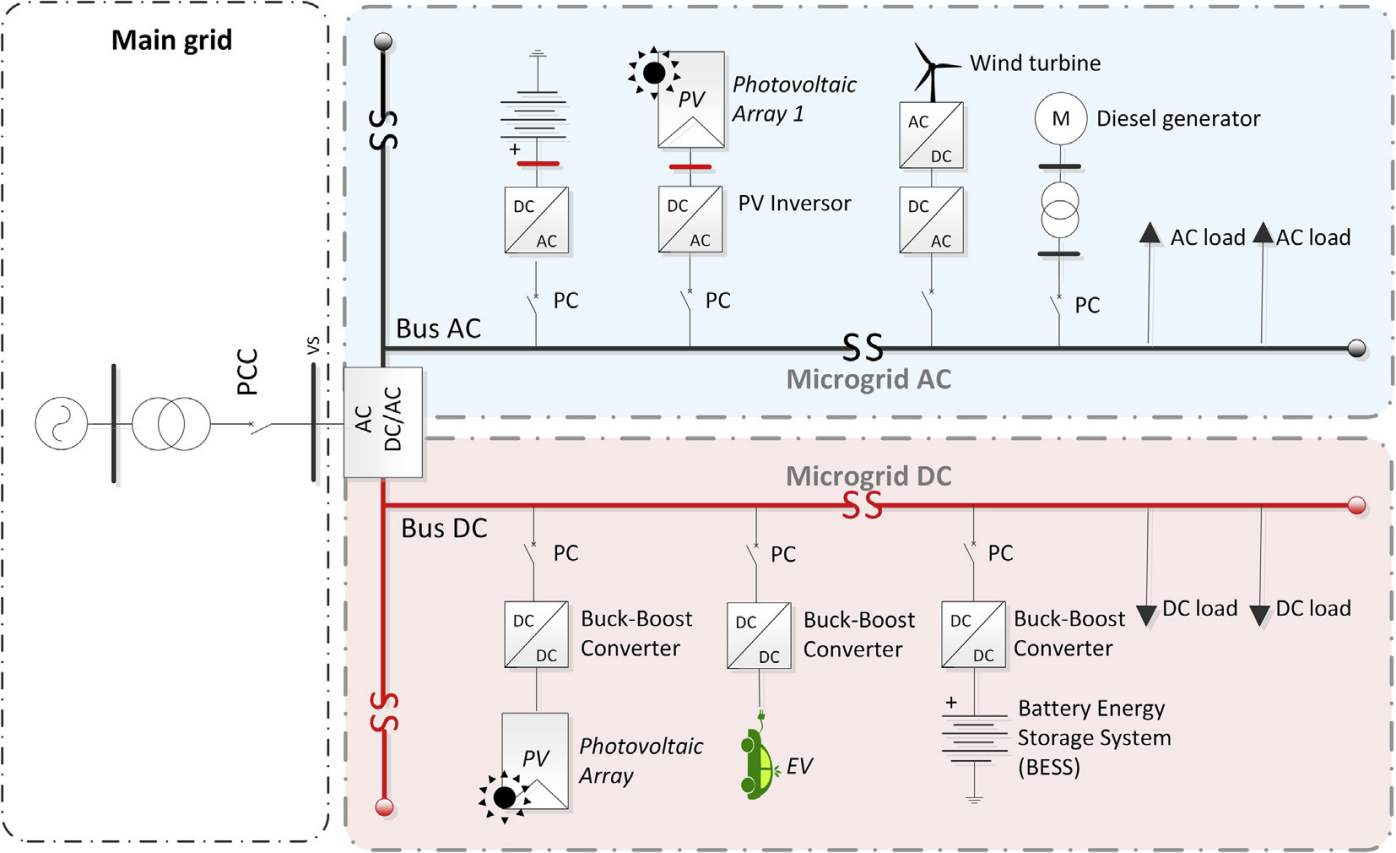
AC/DC HMG.

As shown in Fig. 3, the combination between both configurations gives rise to the concept of AC/DC HMGs while proposing an improved approach that combines the main advantages of the AC and DC MGs [4, 20]. Future trends show a greater requirement and effort in research of characteristics such as: scalability, identification and modelling, design, and control structures that allow an integration of HMGs to the Main Grid [20, 21].

Some papers focus mainly on AC MGs and DC MG architectures. HMGs may represent an interesting solution in which the possible advantages of AC and DC configurations could be combined. The AC MGs can be interconnected (coupled) or isolated (decoupled). Two approaches have been identified in decoupled MGs: fully isolated topology or partly isolated topology.

On the other hand, in decoupled AC three topologies are defined: the completely isolated two-stage configuration, the two partially isolated stages and the configuration of three partially isolated stages. The configuration to be defined for the AC/DC HMG greatly depends on the application and the environment in which it is integrated [4, 5, 20, 21]. The main characteristic of the interconnected configuration is that the AC MG is directly connected to the Main Grid by means of an AC transformer, fixing the MG to the grid [20]. The transformer is located at the point of connection with the electrical grid providing galvanic isolation to the entire MG and reducing the voltage level for the low voltage AC grid. The interconnected HMGs are also capable of managing bidirectional power flow (generation/consumption) using bidirectional AC/DC VSC for the case of BESS storage systems. It is important to emphasize that HMGs are easily scalable and their implementation can be done in several levels and configurations. Additionally, HMGs can be integrated into a medium-voltage (MV) Distribution Grid or in a low-voltage (LV) residential level [20].

When designing an AC/DC HMG, it is necessary to consider some requirements that must be met, such as reliability, controllability, observability, economy and flexibility. The following design principles from literature are taken into account [5].

• The principle of partition*.*
• The principle of hierarchy*.*
• Full use of the resources.
  - *Maximizing the use of resources.*
  - *Energy complementary.*
• Power quality assurance principles.
  - *Storage allocation.*
• Reactive power compensation.
• Configuration: Radial and ring.

### 2.2 Microgrids models and benchmark. A review

The MG models presented in the literature show different characteristics depending on their configuration, type of topology, and components. All of these must deal with dynamics, low energy storage capacity of BESS, high number and diversity of generation, electronic power converters and a high degree of non-linear phenomena [1, 10, 47, 50]. Others seek to model each distributed generation source by reducing the order of the model to a linear and time invariant (LTI) system with a time constant and a gain factor, while neglecting the dynamics of the grid [10, 47]*.*

There are also other approaches with inverter-based DG, for example, in [51] the complete dynamic model of the entire grid was considered in place of the inverter, dividing the MG system into three subsystems: inverter, grid and loads. For the inverter model, the dynamics of the controller, the output filter and the coupling inductor are incorporated. In another paper the authors also develop a model in the LTI linear state space and perform an eigenvalue analysis to study the dynamic behavior of the MG [52]. In [7, 8, 9, 10, 11, 12, 13, 14, 15] real MGs have been implemented all over the world. Also, the authors show a summary of some existing test systems where they include simulated grids and present a comparative summary of them.

## 3 Proposed model

This section presents a detailed description of the proposed three-phase model. It was simulated using the corresponding Simscape library within the MATLAB/Simulink R2017b environment. The aim is to provide a tool for the scientific community and offer a better understanding of the MG dynamics and each of its components, as well as their overall performances under different operating conditions.

**Fig. 4:**
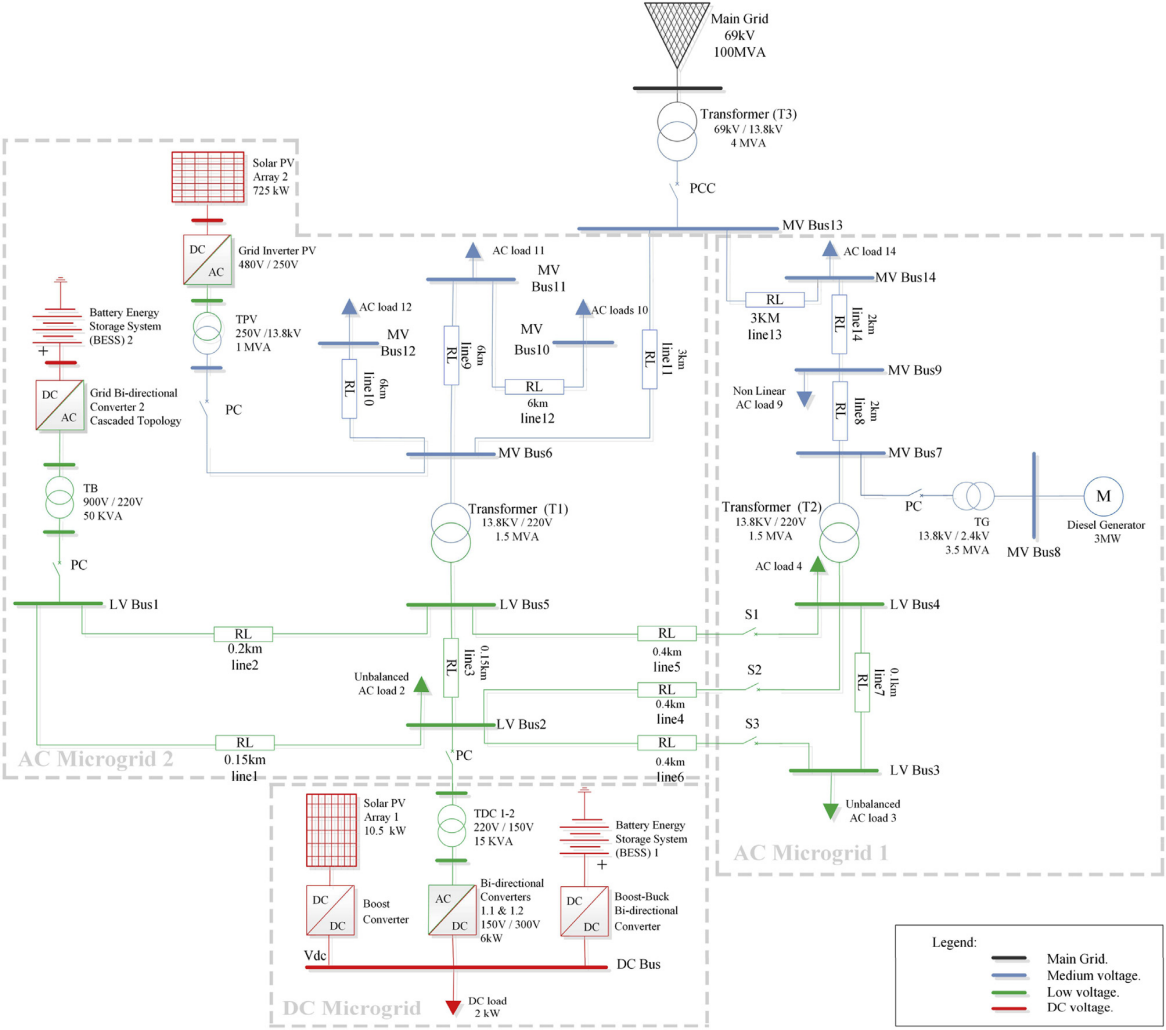
Proposed MG.

Fig. 4 shows a one-line diagram for the proposed benchmark. In this figure, the MG is coupled to the PCC point to a 69-kV electrical sub-transmission system. This utility grid has a Thevenin equivalent of 100 MVA with an X/R ratio of 10. There are two voltage distribution levels: a primary 13,8-kV voltage level, depicted in blue color and a secondary 0,22-kV voltage level depicted in green color. Three sub MGs are shown: AC MG 1 is an area connected at the 0,22-kV level through lines 4, 5 and 6 to the AC MG 2. Switches S1, S2 and S3 are considered connected in this paper. It operates with a diesel generator and provides energy to 4 loads. AC MG 2 is another area, which operates with the PV Array 2 and the BESS 2. Both AC MGs operate at a frequency of 60 Hz. The third area is a DC busbar comprising the BESS 1 and the PV Array 1. The BES system 1 is connected through a boost-buck bidirectional converter, while the PV Array 2 is connected to the DC link through a boost converter. The DC busbar is linked to the AC MG 2 through two parallel bidirectional converters tagged 1.1 and 1.2 which can operate as rectifiers or inverters to exchange active and reactive power through two transformers named TDC-1 and TDC-2. In the one-line diagram, an equivalent bidirectional converter has been placed instead of the two original converters. So is the case of transformer TDC 1–2 which takes the place of both transformers TDC-1 and TDC-2. These simplifications to the one-line diagram are made for the sake of simplicity. Finally, this AC/DC HMG also includes a diesel generator and a Main Grid equivalent system with linear and nonlinear loads.

### 3.1 Solar-Photovoltaic subsystems (PV)

Solar-Photovoltaic technology is one of the distributed renewable energy resources in the MG. Table 1 shows main parameters, which are used by the MATLAB/Simulink environment to characterize performance of the solar cell panels. Both arrays operate with total irradiance on the solar cells of G = 1000 W/m^2^ and a temperature of 25 °C. Array 1 has 42 modules which develops a nominal power of 10,5 kW whereas array 2 has 1750 modules, which develops a nominal power of 725 kW for the DC Bus. Both DC-type sources of energy have need of power converter interfaces. Array 1 operates with a DC-DC voltage-controlled boost converter. It has a 6000 μF link capacitor operating at 300 VDC. A PWM closed-loop control strategy utilizes a frequency of 5 kHz. Array 2 operates with an open-loop sinusoidal PWM inverter. Its m index is set to 1.0 and steps down from 480 VDC to 250 VAC.

**Table 1:** PV arrays for the MG system

ID	Current at maximum power point I_mpp_ (A)	Maximum Power (W)	Open circuit voltage V_oc_ (V)	Voltage at maximum power point V_mp_ (V)	Short-circuit current I_sc_ (A)
Array 1	8,63	250	37,4	30,7	8,63
Array 2	5,69	414,8	85,3	72,9	6,09

### 3.2 Battery energy storage systems (BESS)

BESS #1 operates in the DC bus. It consists of 1 lithium-ion battery unit of 120 VDC nominal voltage with a rated capacity of 800 (Ah). BESS #2 operates near LV Bus 1. It consists of 3 nickel-metal-hydride (Ni–MH) battery units of 650 VDC nominal voltage each one with a rated capacity of 1,5 (Ah). The parallel-connected batteries are connected to an interfaced inverter in a cascaded topology which in turn steps up from 650 VDC to 900 VAC. All these battery-types are devices available in the Simscape library within the MATLAB/Simulink environment.

The Simscape library integrates Eqs. (1), (2), (3), and (4) which govern the discharge-charge process of the nickel-metal-hydride batteries [53]:

(1) $$E_{disch}^{\text{Ni}–\text{MH}}=Eo-k\frac{Q}{Q-it}i^{*}-k\frac{Q}{Q-it}it+Exp\left(t\right)$$

(2) $$E_{ch}^{\text{Ni}–\text{MH}}=Eo-k\frac{Q}{\left|it\right|-0.1Q}i^{*}-k\frac{Q}{Q-it}it+Exp\left(t\right)$$

The discharge condition implies $i^{*}>0$ while the charge condition implies $i^{*}<0$. On the other hand, the equations that govern the discharge-charge process of the lithium-ion batteries are:

(3) $$E_{disch}^{\text{Li}-\text{Ion}}=Eo-k\frac{Q}{Q-it}i^{*}-k\frac{Q}{Q-it}it+AExp\left(-Bit\right)$$

(4) $$E_{ch}^{\text{Li}-\text{Ion}}=Eo-k\frac{Q}{\left|it\right|-0.1Q}i^{*}-k\frac{Q}{Q-it}it+AExp\left(-Bit\right)$$

The discharge-charge conditions are the same as those of the Ni–MH battery type. In the equations, the variables and parameters are:

***E***_***o***_ is the battery constant voltage;
K is the polarization constant (Ah−1);
Exp (t) is the exponential zone dynamics, in V;
Q is maximum battery capacity, in Ah;
it is extracted capacity or the actual battery charge, in Ah;
i* is the filtered low frequency current dynamics, in A;
A is exponential voltage, in V;
B is exponential capacity, in (Ah)^−1^.

**Fig. 5:**
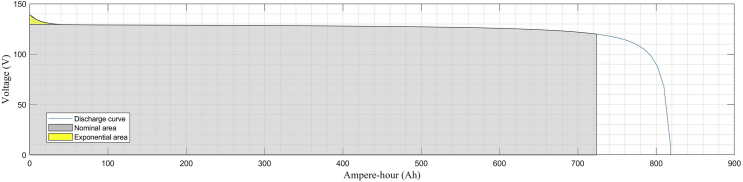
Nominal current discharge curves for the lithium ion battery (BESS #1) at 0.43C (347 A).

**Fig. 6:**
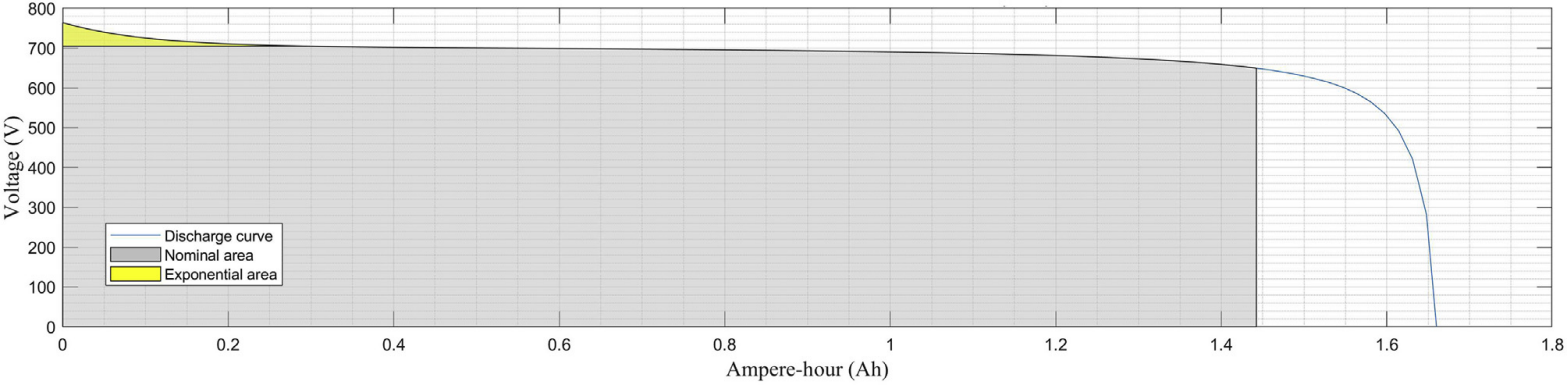
Nominal current discharge curves for one nickel-metal-hydride (Ni–MH) battery (BESS #2) at 0.2C (0.3A).

Fig. 5 shows the typical constant current/discharging voltage characteristic curve for the lithium-ion battery. Fig. 6 shows the corresponding curve for the nickel-metal-hydride battery. For both curves, three regions are clearly distinguishable: the exponential region in yellow, the nominal or rated region colored in gray and the final discharge behavior in blue [54].

### 3.3 DC-AC voltage source converters

The solar PV array #2 is interconnected to the AC 13,8 kV system through an inverter which steps down from 480 VDC to 250 VAC. Transformer TPV is used to connect the AC inverter side to the MG primary distribution system. The converter is modelled using a 3-level PWM-controlled IGBT bridge. The PWM carrier frequency is 1980 Hz operating at a closed-loop control strategy for both scenarios.

The BESS #2 is also interconnected to the AC 0,22 kV system with an inverter which steps up from 650 VDC to 900 VAC. Transformer TB is used to connect the AC inverter side to the MG secondary distribution system. The inverter is modelled using a multilevel topology determined to be the most efficient and reliable, considering power losses, total harmonic distortion and efficiency. In a recent paper, five different topologies were contrasted and the CMLI (Cascade h bridge Multi Level Inverter) was chosen [55].

A phase shifted SPWM technique is used in an open loop strategy with a carrier frequency of 2500 Hz. In the maximum demand scenario, the inverter generates to the AC inverter side using an overmodulation index of 1,2 whereas in the minimum demand scenario, the inverter can charge the BESS #2 unit from the AC inverter side using a modulation index of 0,8.

### 3.4 Transformers proposed for the microgrid system

There are 7 power transformers in the main case study. A 69 kV/13,8 kV substation transformer, named T3, has a series equivalent impedance of 1,5% on its MVA base and has a delta-wye grounded configuration. It connects the distribution MG to the 69 kV sub-transmission system. The upstream utility grid has a Thevenin equivalent of 100 MVA with an X/R ratio of 10.

Transformers T1 and T2 are used to step down from the 13,8-kV industrial or commercial usage to the 0,22-kV residential usage. On the other hand, transformers TB, TG, TPV and TDC1-2 are used with the energy storage systems and the renewables to interface with the inverters from the MG. A step-up transformer, named TG, connects the distribution diesel generator to the grid. A complete detail of transformers and their ratings are shown in Table 2.

**Table 2:** Transformer ratings for the MG system.

Transformer	Nominal Power (kVA)	Voltage Ratio (HV/LV)	R_cc_ (pu)	X_cc_ (pu)
T1	1500	Y 13800/220 Y	0,03	0,03
T2	1500	Y 13800/220 Y	0,03	0,03
T3	4000	Yg 69000/13800 D1	0,015	0,015
TB	55	D1 900/220 Y	0,003	0,06
TG	3500	Yg 13800/2400 D1	0,015	0,015
TPV	1000	Yg 13800/250 D1	0,0012	0,03
TDC1-2	15	Y 220/150 Y	0,03	0,06

### 3.5 Line data

Distribution lines for the benchmark MG are typical in AC medium and low voltage distribution levels. For the 13,8-kV primary grid, a 1/0 Cu bare conductor was chosen. The unit resistance of the line is 0,394 Ω/km, the unit reactance of the line is 0,1168 Ω/km and the unit impedance of the line is 0,411 Ω/km. For the voltage level 0,22-kV secondary grid, a 4/0 Cu TW cable was chosen. The unit resistance of the line is 0,198 Ω/km, the unit reactance of the line is 0,1089 Ω/km and the impedance unit of the line is 0,227 Ω/km Table 3 gives more details:

**Table 3:** Line data for the MG system.

Line	Sending end	Receiving End	R (ohm)	X (ohm)	Distance (km)
1	LV1	LV2	0,0297	0,016335	0,15
2	LV1	LV5	0,0396	0,02178	0,2
3	LV2	LV5	0,0297	0,016335	0,15
4	LV2	LV4	0,0792	0,04356	0,4
5	LV4	LV5	0,0792	0,04356	0,4
6	LV2	LV3	0,0792	0,04356	0,4
7	LV3	LV4	0,0198	0,01089	0,1
8	MV7	MV9	0,788	0,2336	2,0
9	MV6	MV11	2,364	0,7008	6,0
10	MV6	MV12	2,364	0,7008	6,0
11	MV6	MV13	1,182	0,3504	3,0
12	MV10	MV11	2,364	0,7008	6,0
13	MV13	MV14	1,182	0,3504	3,0
14	MV9	MV14	0,788	0,2336	2,0

### 3.6 Loads

Table 4 shows the data loads connected to the system, its power factors and unbalanced voltage percentages. Balance and unbalance linear loads are modelled as constant impedances. Loads connected to buses 2 and 3 contain single-phase components that deteriorate the symmetry of voltages and currents in this system. The unbalanced percentage of the loads can be calculated as the percentage value between the maximum deviation of the current of one of the phases with respect to the average load current, as shown in (5) and (6).

(5) $$UL\%=\frac{max\;\left\{\left|I_{a}-I_{average}\right|\;\;;\;\left|I_{b}-I_{average}\right|\;\;;\;\left|I_{c}-I_{average}\right|\right\}\;}{I_{average}}*100$$

(6) $$I_{\mathrm{average}}=\frac{I_{a}+I_{b}+I_{c}}{3}$$

where:

$I_{a},\;I_{b},\;I_{c}$ are the line rms currents in phases a, b and c.

**Table 4:** Load data for the MG System.

Bus ID	Load Name	Load Type	Max. load (kVA)	Min. load (kVA)	PF	Unbalance load (%)
LV2	Load 2	Unbalanced load	40	12	0,9	13
LV3	Load 3	Unbalanced load	30	9	0,85	12,6
LV4	Load 4	Linear load	50	15	0,9	0
MV9	Load 9	Non-linear load	320	96	1	0
MV10	Load 10	Linear load	800	240	0,8	0
MV11	Load 11	Linear load	400	120	0,8	0
MV12	Load 12	Linear load	800	240	0,8	0
MV14	Load 14	Linear load	1600	480	0,8	0
DC	DC load	-	2	0,6	1	-

### 3.7 Non-linear load

A three-phase SCR bridge rectifier operates as a non-linear load at MV Bus9. In the maximum demand condition, an open-loop PWM strategy, 4080 Hz carrier frequency with an m_index_ of 0,8 defines the operation of the converter with a DC load of 1084 Ω. In the minimum demand condition, with unaltered PWM parameters, a DC load of 3615 Ω is placed instead. The rest of parameters are shown in Table 5.

**Table 5:** Data for the non-linear Load.

PWM Freq. (Hz)	Capacitor (μF)	V_n_ (V_dc_)	R [P_max_] (Ω)	R [P_min_] (Ω)	m_index_ -	phi (°)
4080	10	18500	1084	3615	0,8	-30

### 3.8 DC bus (PV, BES systems, DC/DC and DC/AC VSC)

**Fig. 7:**
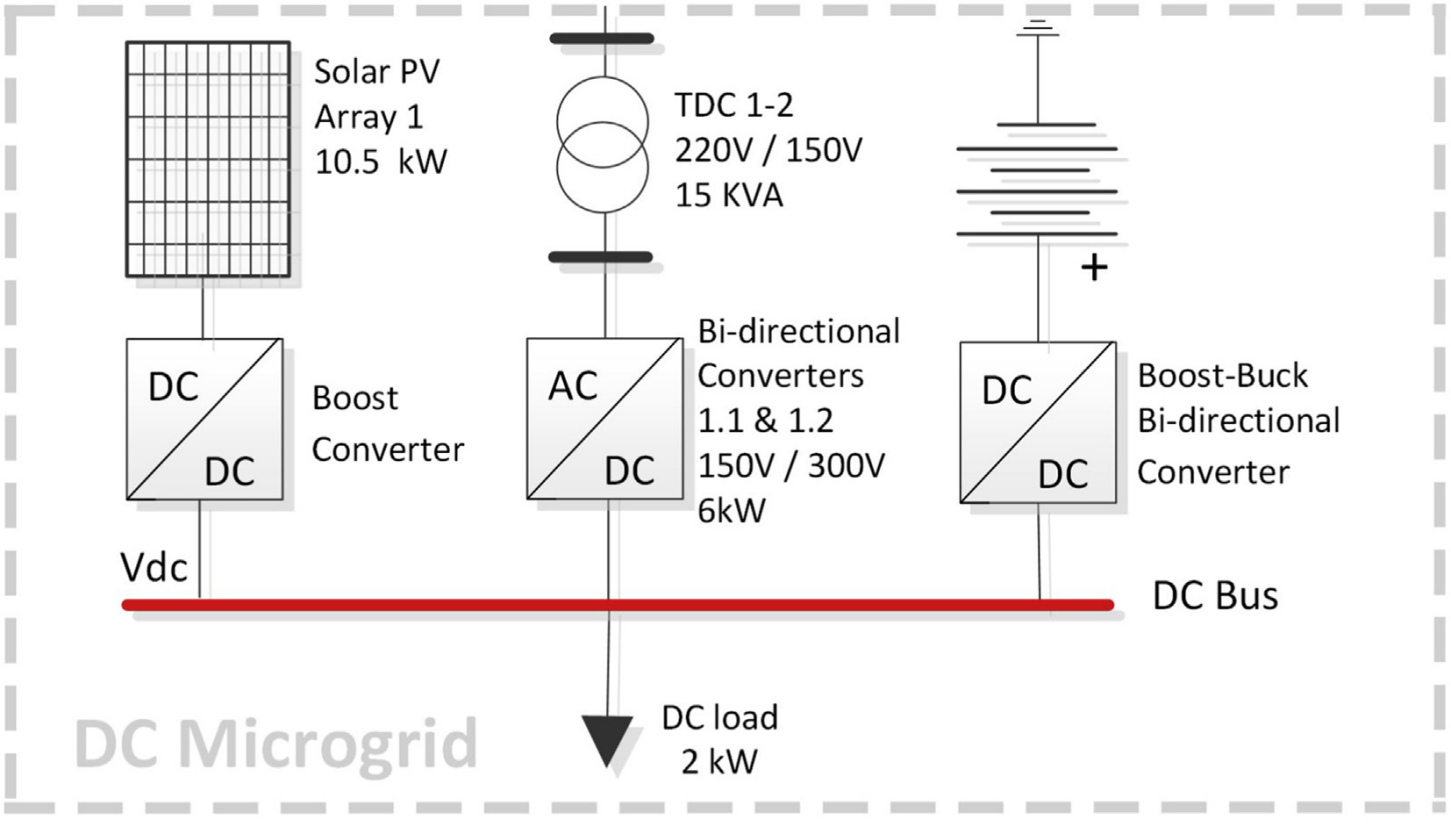
DC MG.

As shown in Fig. 7 and explained below, the DC MG bus operates in two opposite situations: a scenario of maximum and minimum demand. In the first operating mode (maximum demand), the PV-1 system can operate to track the maximum power point (maximum power point tracking control - MPPT), and therefore, the AC MG functions as an energy storage of a certain capacity. In the second mode (minimum demand), there is no power generation in the PV-1 system, so the link to the AC MG #2 through the bidirectional AC/DC VSC control the power flow from the AC MG #2 to the DC MG, allowing the storage of energy in the BESS #1 and supply the load in the DC system.

The DC bus has a 2-kW load in the maximum demand scenario. It was modelled as a linear resistance. In future investigations, it can be replaced with a charging station model for electric vehicles (plug-in hybrid electric vehicle, PHEV or electric vehicle, EV). In this perspective, with a SG deployment, the vehicle to grid (V2G) can also become an agent that operates as an energy provider [19, 37, 56].

The DC bus converters were modelled as a bidirectional DC/DC converter for the BESS #1 and a DC/DC amplifier controller for the PV #1. Details of this DC MG are given in Table 6. This arrangement was inspired by the model proposed in [57].

**Table 6:** DC converter parameters and specifications.

DC Bus Converter	PWM Frequency (Hz)	Capacitor (uF)	V_n_ (V_dc_)
Bidirectional Boost-Buck	5000	1200	300
Boost	5000	1200	300

Output voltages from the distributed generation sources, as PV systems, are typically intermittent by nature. These systems are totally dependent on random phenomena such as changes in irradiation and temperature that depend on weather conditions during the day. Therefore, it is essential to connect both to the DC bus through a controlled DC-DC converter in order to regulate the output voltage or to follow the maximum power point (MPPT). A topology was chosen for the DC/DC boost converter that is intended to increase the input DC voltage (V_i_) in a range of 100 V–130 V up to a DC output voltage (V_o_) about 350 V, which is the DC bus voltage level, as recommended by the authors in [57]. The switching frequency *f* is set to 5000 Hz in order to avoid two typical problems: for one thing, the audible noises and for another to avoid the high-frequency parasitic elements. To maintain the controller stability, both parameters are selected above the critical values already discussed in [57, 58]. These critical values are given by Eqs. (7) and (8):

(7) $$L_{c}=\;\frac{D\;\left(1-D\right)}{2f}\;R$$

(8) $$C_{c}=\;\frac{D}{2fR}$$

where:

$L_{c}$ , is the inductance critical value;
$C_{c}$, is the capacitance critical value;
D , is the large-signal duty cycle;
f , is the switching frequency;
*R* , is the load resistance.

In this case, capacitance is set to 1200 μF and the inductance is set to 9 mH, considering a DC resistance of 45 Ω which corresponds to a power consumption of 2 kW at nominal voltage.

In order to control the loading and unloading processes of the storage device BESS #1, another bidirectional DC-DC controller interfaces two voltages, the DC bus voltage and the battery system voltage. Two PI controllers are intended to achieve the reference current signal in case of loading and unloading. The controller follows a current reference signal to charge and discharge the BESS #1, which is connected to the DC MG. The aim is to target low current ripples in order to achieve greater efficiency and increase the useful life of the battery system. It would also be useful to know that when the MG goes into isolated mode, the control strategy regulates the DC bus voltage [57].

Eqs. (9), (10), (11), and (12) are considered in order to select the filter values. BESS batteries are simulated according to characteristics presented in Table 7, Figs. 5 and 6.

(9) $$\Delta I_{HV\;side}=\;\frac{V_{i}}{fL}\;D$$

(10) $$\Delta V_{HV\;side}=\;\frac{I_{o}}{fC}\;D$$

(11) $$\Delta I_{LV\;side}=\;\;\frac{V_{o}\;\left(V_{i}-\;V_{o}\right)}{fL\;V_{i}}\;D$$

(12) $$\Delta V_{LV\;side}=\;\;\frac{V_{i}}{8\;L\;C\;f^{2}}\;D\;\left(1-D\right)$$

where:

$\Delta I_{HV}$ , the inductor current for the boost side;
$\Delta V_{HV}$, the capacitor voltage ripple for the boost side;
$\Delta I_{LV}$ , is the inductor current for the buck side;
$\Delta V_{LV}$ , is the capacitor voltage ripple for the buck side.

**Table 7:** BESS systems for the MG distribution system.

ID	Battery Unit	Nominal Voltage (V)	Rated Capacity (Ah)	Initial SOC (%)
BESS # 1	Battery 1	120	800	80
BESS # 2	Battery 1	650	1,5	80
	Battery 2	650	1,5	80
	Battery 3	650	1,5	80

There is no doubt that bidirectional AC/DC converters have many applications in the microgrid field. Therefore, the power interaction between the DC bus and the AC bus (see Fig. 7), was proposed in this study using two bidirectional converters of several switching states for the inversion or rectifier modes. For this conversion system, a three-phase universal power module is implemented. It consists of six power switches connected in a bridge configuration, by means of electronic power devices such as diodes and IGBTS (device-controlled switching).

A configuration of two-paralleled bidirectional AC/DC converters is presented. It is designed to convert the voltage from 300 VDC to an output voltage of 150 VAC. It allows to exchange the power generated in the DC bus to the AC grid. This solution becomes interesting from the point of view of increasing robustness, flexibility, and performance of the Microgrid in DC, which are designed to maximize performance for a wide range of powers. The bidirectional converters were considered with a master-slave control scheme.

The input voltage (*V*_*input*_) and output voltage (*V*_*output*_) are the same for the two conversion modules, just as the input current (*I*_*input*_) and output current (*I*_*output*_) are the result in the sum of the currents in each individual module, see Eqs. (13) and (14).

So,

(13) $$I_{input}=\;\;\overset{n}{\underset{j=1}{\sum }}I_{input,\;\;j}\text{ and }I_{output}=\;\;\overset{n}{\underset{j=1}{\sum }}I_{output,j}$$

(14) $$P_{input}=\;\;\overset{n}{\underset{j=1}{\sum }}P_{input,\;j}\text{ and }P_{output}=\;\;\overset{n}{\underset{j=1}{\sum }}P_{output,\;j}$$

where:

n is the total number of modules (for this case n = 2);
$I_{\mathrm{input},\;j}$ is the input current of the jth module;
$P_{\mathrm{input},j}$ is the input power of the jth module.

## 4 Power flow results

The AC/DC MG test system model as shown in Fig. 4, is simulated using Simscape within Simulink. In order to obtain a better software performance during the simulation process, a step time of 5 μs is chosen. Values for all angle buses are computed by using a PLL-based adaptive notch filter [59]. The Phase-Locked Loop auxiliary circuit extracts the phase angle from the instantaneous voltage waveform of one phase as well as the phase angle from the instantaneous current waveform of the same phase. Then, a subtraction is made of these two values to compute φ for every bus. Further details of the PLL design and its performance are shown in [59].

The power block computes the active power (P_i_) and the reactive power (Q_i_) of a voltage-current pair signal at the fundamental frequency. In order to perform this computation, the block determines the peak magnitude of the two input signals V_i_(t) & I_i_(t), |V_i_| and |I_i_|. The output variables (P_i_) and (Q_i_) are then calculated in every period of the fundamental waveform, as shown in Eqs. (15), (16), and (17):

(15) $$\varphi_{i}=\;∠V_{i}-∠I_{i}$$

(16) $$P_{i}=\;\;\frac{\left|V_{i}\right|}{\sqrt{2}}\;\frac{\left|I_{i}\right|}{\sqrt{2}}\;cos\;\left(\varphi_{i}\right)$$

(17) $$Q_{i}=\;\;\frac{\left|V_{i}\right|}{\sqrt{2}}\;\frac{\left|I_{i}\right|}{\sqrt{2}}\;sin\;\left(\varphi_{i}\right)$$

where:

$\left|{\text{V}}_{\text{i}}\right|$ is the peak voltage value for one phase;
$\left|{\text{I}}_{\text{i}}\right|$ is the peak current value for the same phase;
${\text{φ}}_{\text{i}}$ is the difference between the voltage angle and current angle for the same phase;
${\text{P}}_{\text{i}}$ is the per-phase active power;
${\text{Q}}_{\text{i}}$ is the per-phase reactive power;
i  is the bus number.

This section reports on the simulation results for the proposed test system. The MG keeps its operation stable at steady state for both conditions: maximum and minimum demand scenarios. Although the calculations furnished by Simulink are per phase, active and reactive power values shown on Tables 8 and 9 are the sum of the per-phase power that is, three-phase active power and reactive power, respectively. For any generator bus, P_g_ and Q_g_ are presented, whereas for any load bus, P_l_ and Q_l_ are presented. Lastly, any transfer bus, P_transf_ and Q_transf_ are shown.

**Table 8:** Load flow results for the maximum demand scenario.

Bus *i*	Type	P_g_ (kW)	Q_g_ (kVAr)	P_l_ (kW)	Q_l_ (kVAr)	P_transf_ (kW)	Q_transf_ (kVAr)	V (pu)	δ (°)
LV 1	BESS 2	42,66	30,45	-	-	-	-	0,955	-29,76
LV 2	Transfer Bus	-	-	-	-	41,38	4,91	0,931	-30,76
LV 3	Transfer Bus	-	-	64,72	40,11	62,6	43,86	0,930	-31,26
LV 4	Transfer Bus	-	-	-	-	120,66	84,61	0,953	-31,5
LV 5	Transfer Bus	-	-	-	-	34,73	58,77	0,951	-31,25
MV 6	Transfer Bus	-	-	-	-	780	1095	0,966	-30,31
MV 7	Transfer Bus	-	-	-	-	554,6	356,2	0,971	-30,72
MV 8	Diesel	690	450	0	0	-	-	0,975	-60,84
MV 9	Non-linear load	-	-	327,3	38,23	-	-	0,966	-30,67
MV 10	-	0	0	572,4	427,2	-	-	0,94	-29,81
MV 11	-	-	-	290,28	217,71	-	-	0,953	-30,01
MV 12	-	-	-	586,2	439,8	-	-	0,957	-30,11
MV 13	Slack	1810,2	1665	0	0	-	-	0,974	-30,66
MV 14	Transfer Bus	-	-	119,61	89,7	226,02	397,2	0,967	-30,59
DC	DC Bus	-	-	-	-	8	0	0,928	0

**Table 9:** Load flow results for the minimum demand scenario.

Bus	Type	P_g_ (kW)	Q_g_ (kVAr)	P_l_ (kW)	Q_l_ (kVAr)	P_transf_ (kW)	Q_transf_ (kVAr)	V (pu)	δ (°)
LV 1	BESS 2	27,6	15,75	-	-	-	-	0,9567	-31,27
LV 2	Transfer Bus	-	-	-	-	12,64	5,77	0,959	-31,28
LV 3	Transfer Bus	-	-	21,1	13,09	25,47	14,43	0,967	-31,14
LV 4	Transfer Bus	-	-	-	-	50,3	25,74	0,979	-31,14
LV 5	Transfer Bus	-	-	-	-	51	26,79	0,983	-31,24
MV 6	Transfer Bus	-	-	-	-	471,2	346,1	0,980	-30,31
MV 7	Transfer Bus	-	-	-	-	89	84,4	0,98	-30,25
MV 8	Diesel	150,1	49	0	0	-	-	0,98	-60,23
MV 9	Non-linear load	-	-	110,2	0,69	-	-	0,98	-30,28
MV 10	-	0	0	183	135	-	-	0,97	-30,15
MV 11	-	-	-	92,1	69	-	-	0,97	-30,21
MV 12	-	-	-	184,8	138,6	-	-	0,98	-30,24
MV 13	Slack	91,8	74,1	0	0	-	-	0,99	-30,39
MV 14	Transfer Bus	-	-	372	279	21,84	84,6	0,98	-30,3
DC	DC Bus	-	-	-	-	0,975	0	0,985	0

## 5 Simulation analysis of the proposed model

In this section, the reported results are analyzed and metrics of the quality and efficiency variables of the power will also be established. To compute metrics that allow defining a base case for future optimization and compensation studies, two analysis scenarios are defined.

**Fig. 8:**
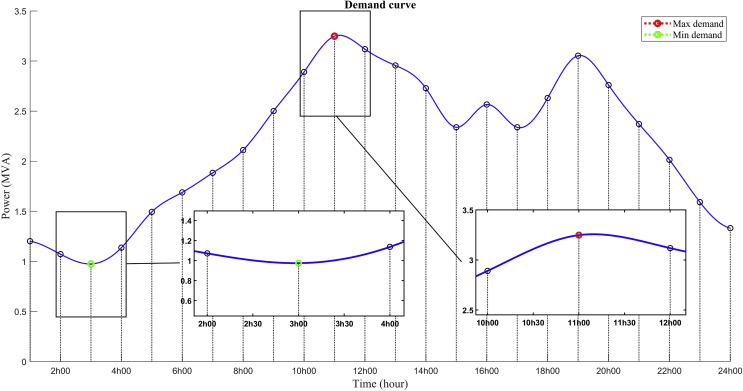
Demand curve. As previously stated, the demand for electrical energy was defined and modelled for two scenarios for which the following dynamic points of operation shown in Table 10 were taken into account.

The daily demand curve of the proposed MG is shown in Fig. 8, which is obtained as the sum of the hourly demands of each load of the model with predominant components of industrial and commercial loads. The loads were modelled with constant impedance and for this reason, a slight difference between the load nominal power and the actual power consumed can be determined. This is due to the voltage drops in the system.

In the daily total demand curve shown in Fig. 8, the minimum demand of the proposed system is approximately 30% of the maximum demand, and this same relation between the active power demands was considered in the total reactive power of the system.

This remarkable difference between the two scenarios allows a real future analysis to be established in terms of reactive power compensation. The capacity and location of the compensating devices must be determined considering the minimum demand scenario. In fact, the voltage profile of the system could reach overvoltage levels with the reactive injection of the compensating devices.

In Fig. 8, it can also be seen that the maximum demand period is at 11h00, which coincides with the peak of solar radiation and, therefore, with the maximum generation capacity of the solar panels. This demand peak during this time is due to the integration of industrial load curves with residential load curves.

**Table 10:** Generations modes for both demand scenarios.

Generations	Maximum demand	Minimum demand
Main Grid	Connected	Connected
Solar PV Array 1 & 2	Generating at 1000 W/m^2^	OFF at 0 W/m^2^
BESS 1	Charge at 5A	Charge at 3A
BESS 2	Generated	Charge
Bidirectional Converter 1.1 & 1.2	Inverter	Rectifier
Diesel Generator	Generating	Generating

### 5.1 Voltage profile analysis

**Fig. 9:**
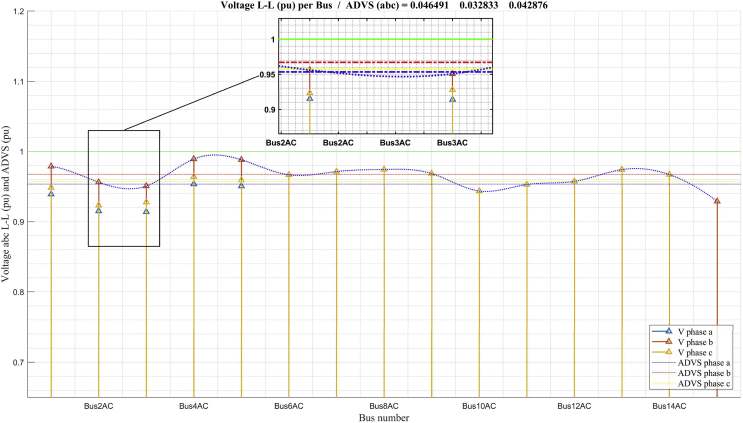
Voltage profile in maximum demand.

**Fig. 10:**
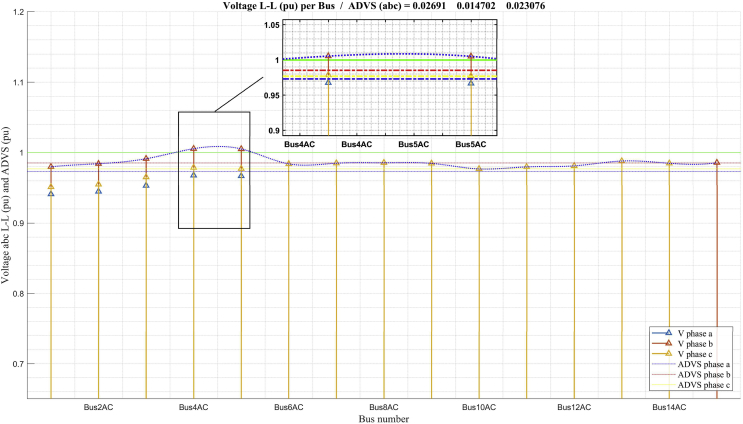
Voltage profile in minimum demand.

Figs. 9 and 10 show the voltage profile results obtained in the scenarios of maximum load power analysis (Fig. 9) and minimum load power (Fig. 10). The analysis of the voltage profiles is shown in phases because the system presents unbalanced loads as a result of single-phase load components. In both cases, it can be seen that the voltage profiles are in permissible operating parameters; however, in maximum demand (Fig. 9) there are significant voltage drops in the low voltage grid (220 V) in buses 2 y 3. This result allows future study for optimal compensation of this indicator. Both figures show indicators that measure the quality of the voltage profile, and the calculation methods used are detailed in Eqs. (18) and (19).

Average deviation of the voltage in the system.

(18) $$ADVS=\frac{\sum_{i=1}^{n}\left|Vd_{i}-V_{i}\right|}{n}$$

Maximum value of voltage deviation.

This function represents the maximum voltage deviation in the system.

(19) $$MVD=\underset{1\leq i\leq n}{\max}\left(\left|Vd_{i}-V_{i}\right|\right)$$

where:

n is the number of buses in the MG;
$V_{i}$ is the real voltage at bus i in p.u. (per unit);
$Vd_{i}$ is the desired voltage at busi in p.u. (1 p.u.);
**ADVS** is a figure of merit that is required to be minimal in a future optimization problem for the improvement of voltage profiles.

Fig. 9 shows that the maximum value of the average deviation of the system voltage in the maximum demand analysis does not exceed 0,046 per unit. However, the maximum value of voltage deviation (MVD) in this system is 0,0691 (phase *a* = 0,0859; phase *b* = 0,0492; phase *c* = 0,0721), and it is in bus 3. This result evidences the need to propose future studies based on finding solutions for the improvement of the voltage profile in this system.

Fig. 10 shows that the voltage profile in the minimum demand scenario presents positive results for all the buses. The average deviation calculations of the system voltage and maximum deviation are very low, which indicates an operation within the desired parameters. This scenario was calculated and analyzed to be considered in reactive power compensation proposals to solve the maximum demand scenario that could affect the voltage profile in the minimum demand scenario.

### 5.2 Active power balance per bus

**Fig. 11:**
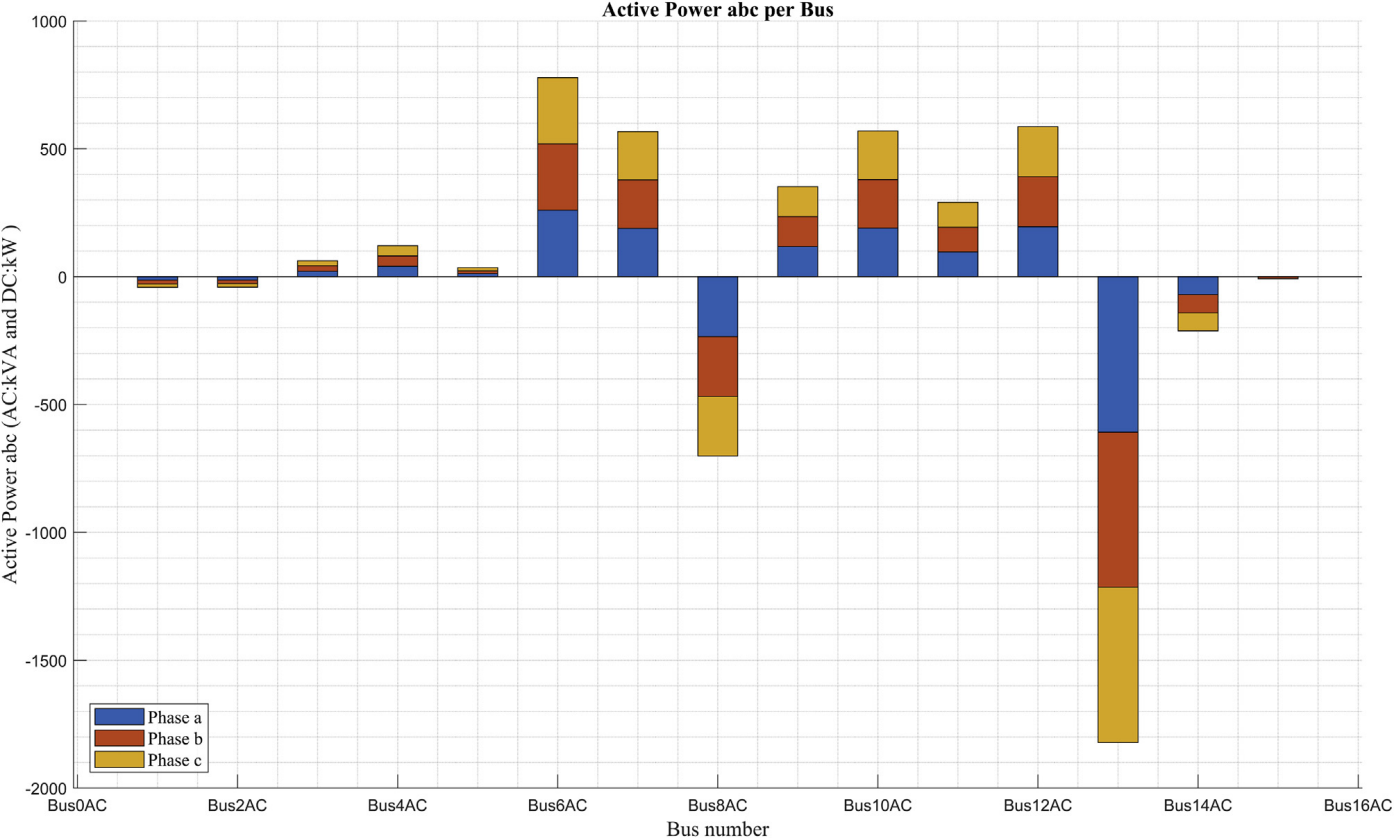
Power balance in maximum demand.

**Fig. 12:**
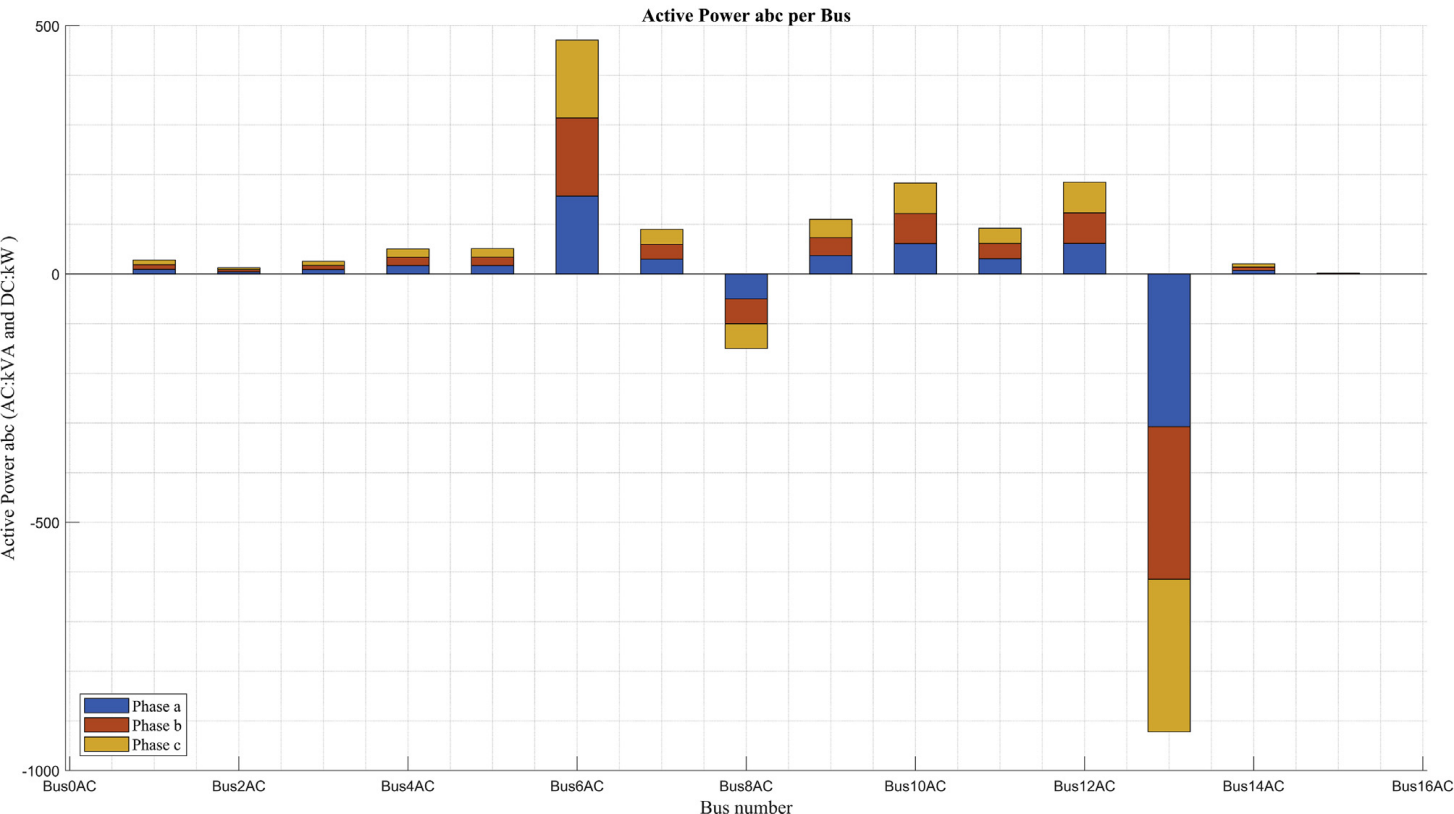
Power balance in minimum demand.

Figs. 11 and 12 show the contribution of the powers in kVA generated and consumed in each of the buses of the system, including the kW power on the DC bus. The positive graphical powers indicate load powers connected in that bus or powers transferred, except in the case of bus 14 in which a negative power is shown, and it is transferred from bus 9 to bus 14 as a power supply contribution of the Diesel generator (this explanation is detailed in Tables 8 and 9) and the rest of negative powers indicate generation in the bus to which corresponds.

In the scenario of maximum demand (Fig. 11) there is a contribution of generation power in 6 buses (1, 2, 6, 8, 13 and DC Bus).

Fig. 12 shows the power balance of the system in the minimum demand scenario, in which the power decreases in each bus with respect to the maximum demand scenario. The contribution in distributed generation for this scenario is also shown, leaving only the Diesel generator connected at bus 8 and the rest of the contribution in active power is delivered by the connected grid through bus 13.

### 5.3 Reactive power balance per bus and angular analysis

**Fig. 13:**
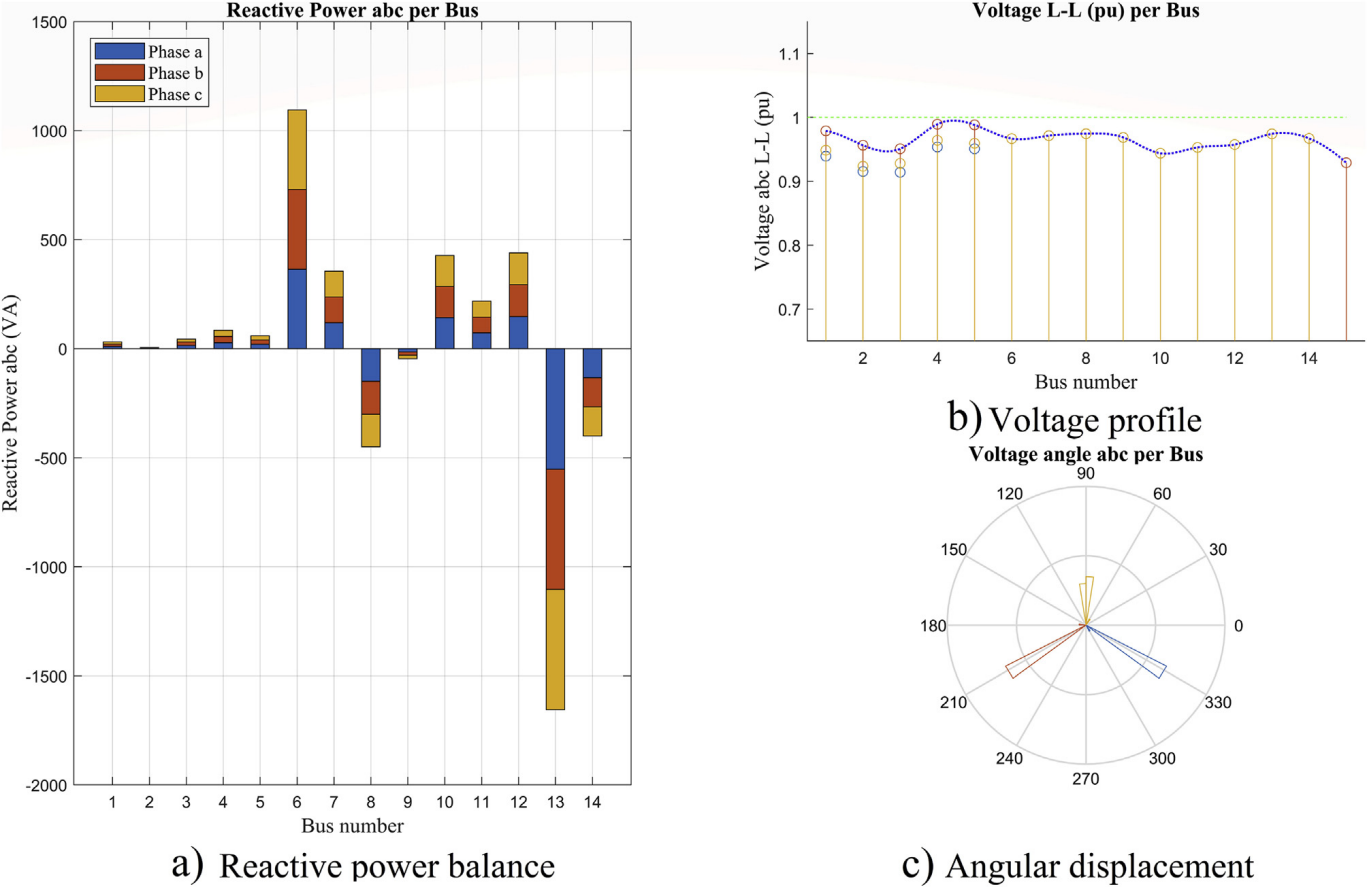
Analysis per bus and phase, in maximum demand scenario, (a) reactive power balance, (b) voltage profile and (c) angular analysis.

**Fig. 14:**
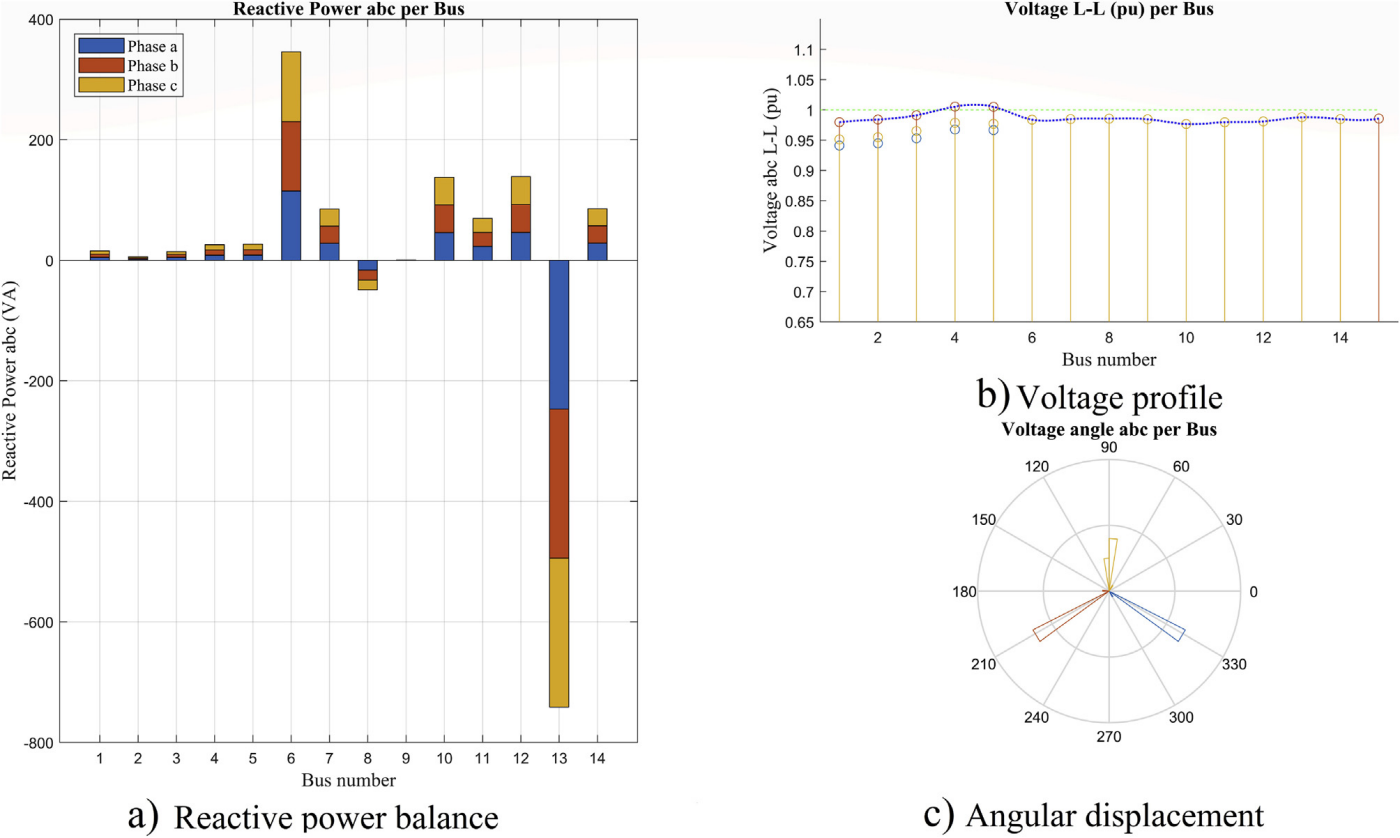
Analysis per bus and phase in minimum demand scenario, (a) reactive power balance, (b) voltage profile and (c) angular analysis.

Figs. 13 and 14 show the reactive power balance per bus, the voltage profile per bus and the variation of phase shift in the voltage angles at the system buses, in maximum and minimum demand respectively. It can be seen that in both figures, there is a coherent relation between the reactive injection and the increase in the voltage profile at the bus where the reactive power is compensated with the existing generation in the system.

The contribution of reactive power in this system is found in bus 8 with the diesel generator and in bus 13 with the reactive power contribution of the external grid. However, in maximum demand (Fig. 13) an apparent injection of reactive power can be seen in buses 9 and 14. Transfer buses show negative powers due to the direction of power flows in this scenario. This explanation is detailed in Tables 8 and 9.

These scenarios provide an important starting point for the analysis of reactive power compensation in order to improve the voltage profiles. The contribution of a minimum demand analysis allows to validate a compensation result in the maximum demand scenario.

Fig. 14 shows the voltage profile in this scenario. It has good values and in some buses, it exceeds 1 p.u. This situation needs a analysis when raising the reactive injection to improve the maximum demand scenario.

### 5.4 Power losses per line

The losses of active power in this system were calculated under conditions of balance and unbalance of currents as defined in [60]. The power losses for a state of charge a on a line b ($\Delta P_{a,b}$), are as indicated in Eq. (20):

(20) $$\Delta P_{a,b}=R_{l}*\overset{Nf}{\underset{i=1}{\sum }}{\left|I_{\;i,a,b}\right|}^{2}+R_{n}*\;\overset{Nf}{\underset{i=1}{\sum }}{\left|I_{\;i,a,b}\right|}^{2}$$

where:

$I_{i,a,b\;\;}$is the current in phase *i* during the state of charge *a,* circulating in line *b*;
$R_{l},\;R_{n}$ are the resistors of the phase and neutral conductors respectively;
Nfis the number of phases of the system in line b.

**Fig. 15:**
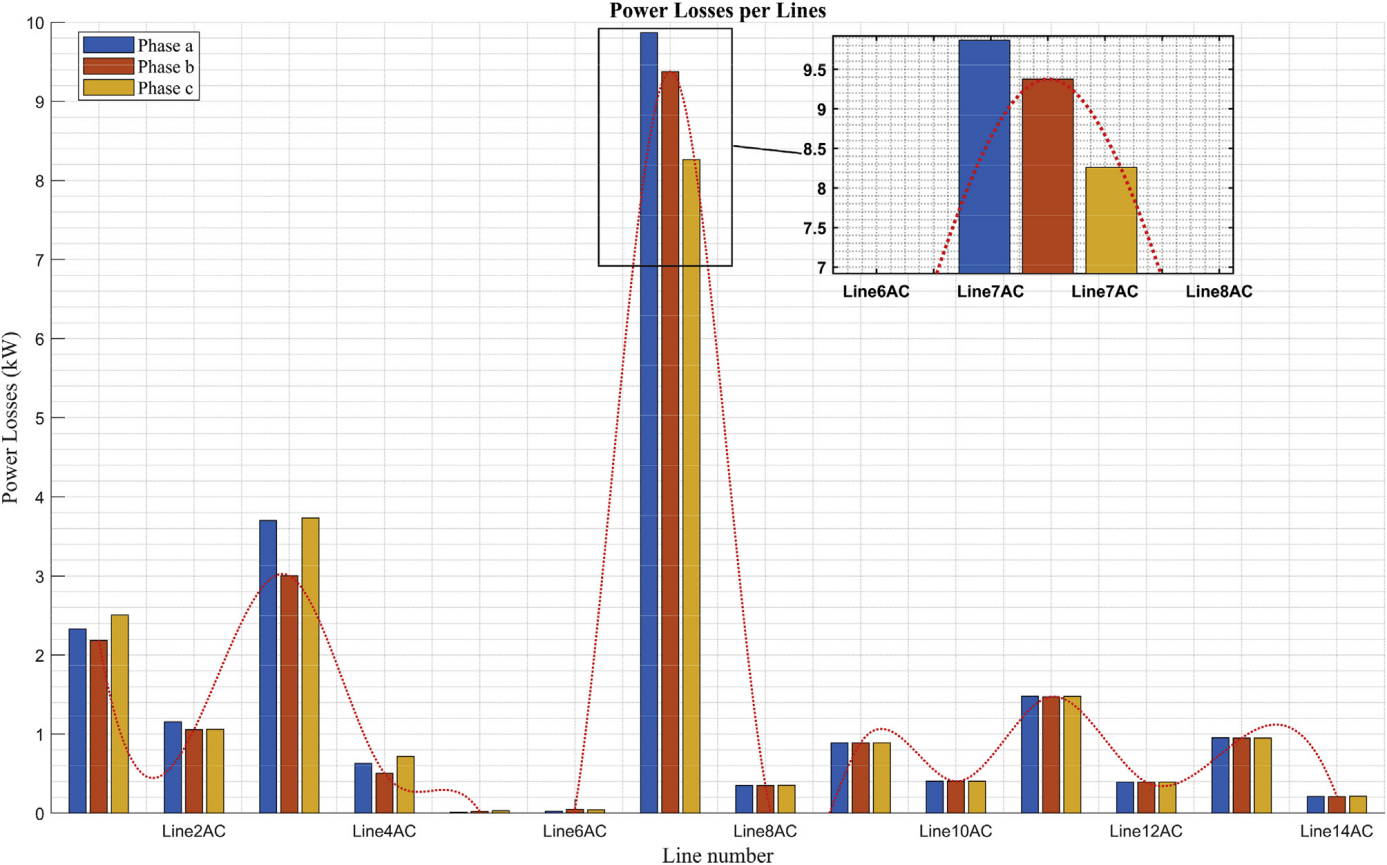
Power losses per line in maximum demand.

**Fig. 16:**
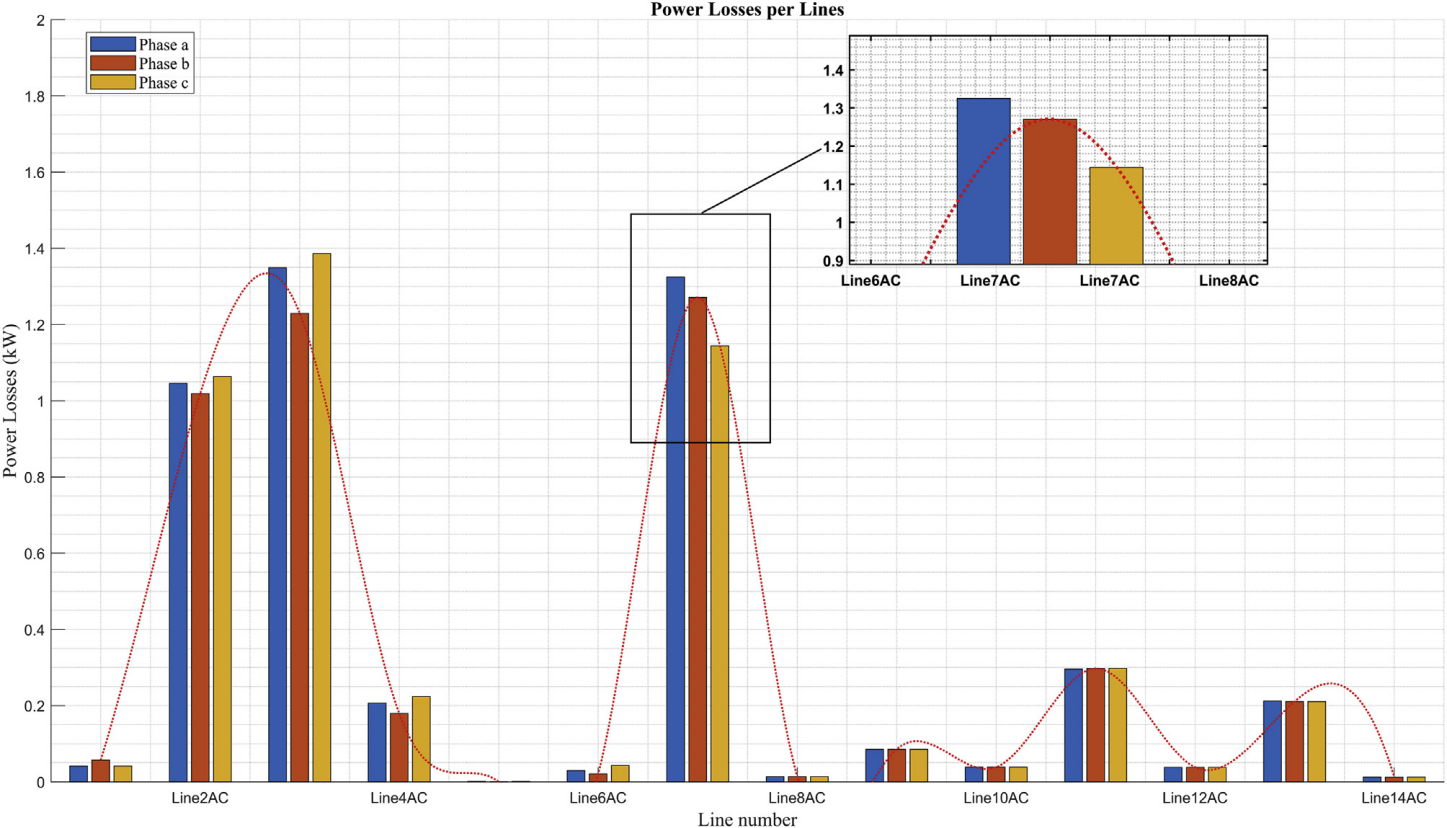
Power losses per line in minimum demand.

Figs. 15 and 16 show the losses per phase in each of the lines of the system, in the scenarios of maximum demand and minimum demand respectively. Both figures show that the highest loss levels correspond to the lines that feed buses in the low voltage grid. It can be seen that the highest level of losses per phase (of almost 9.9 kW) is presented in phase *a* of line 7 (connection between buses 3 and 4), a value that corresponds to the transport of high unbalanced powers in this line, even though its distance is relatively short.

Figs. 15 and 16 show the unbalance of active power losses per phase that exists in the buses with unbalanced loads. This unbalance is due to the component of single-phase loads connected in the low voltage grid of this system.

Fig. 16 shows that the highest level of losses per phase does not exceed 1,4 kW, which is a very low value with respect to the maximum demand losses per phase.

### 5.5 Power factor per bus

**Fig. 17:**
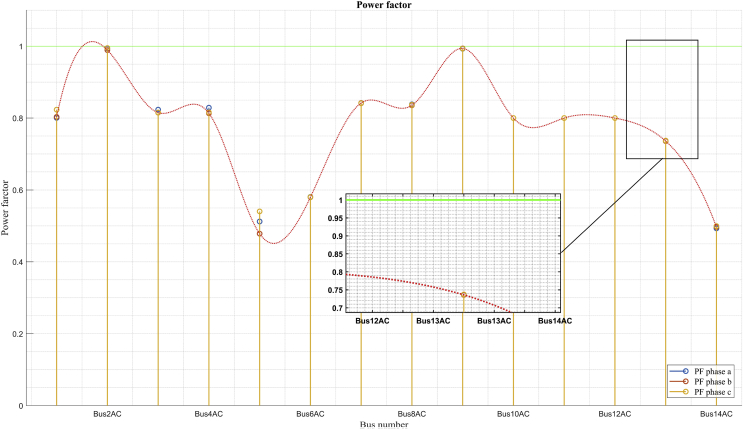
Power factor per bus in maximum demand.

**Fig. 18:**
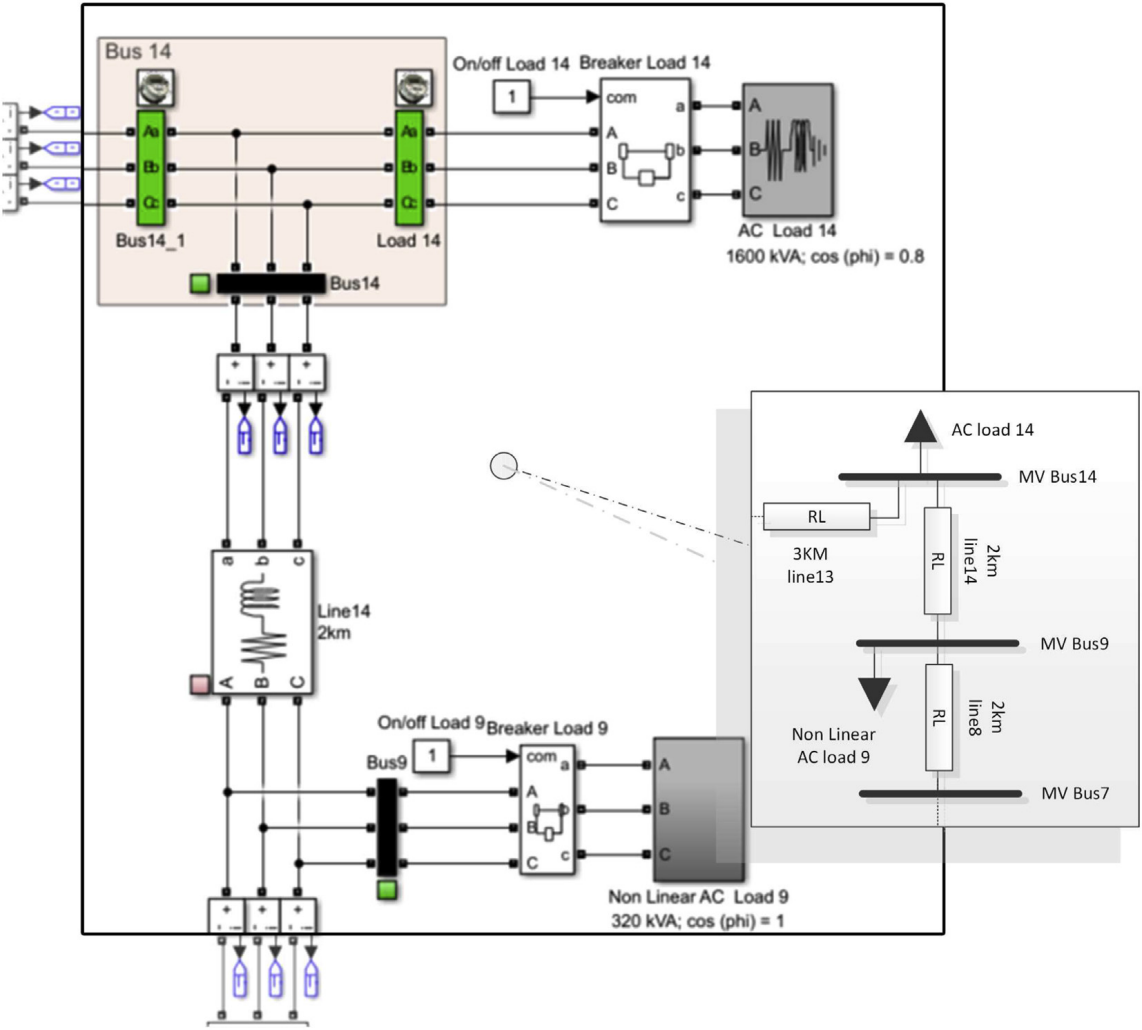
Detail of MATLAB /Simulink topology of bus 9 and 14.

**Fig. 19:**
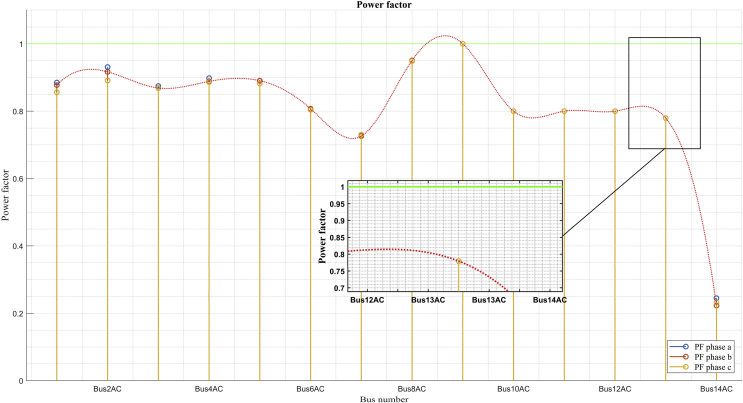
Power factor per bus in minimum demand.

The power factor per bus is one of the most important variables that should be considered in the calculation of power flows in systems which need to be compensated. Figs. 17 and 19 show the power factor for each bus of the system in the scenarios of maximum demand and minimum demand respectively. In the scenario of maximum demand (Fig. 17) it can be seen that the power factor in buses 2 and 9 is almost equal to 1. This is due to the fact that in Bus 2, in maximum demand, there is only a contribution of active power from the DC MG, while the almost unitary power factor in bus 9 is due to the fact that the measurement was made directly in the non-linear load, as shown in Fig. 18. Tables 11 and 12 show the relation between the active and reactive powers in bus 9 corresponding to the non-linear load, in the maximum and minimum demand scenarios.

**Table 11:** Maximum demand power factor for bus 14 and 9: Detail.

Bus	Details	P (kW)	Q (kW)	PF
MV bus 14	Bus 14_1	970	499,8	0,88
	Load 14	1196,1	897	0,8
	MV Bus 14	226,02	397,2	0,49
MV bus 9	MV bus 9	327,3	38,23	0,99

**Table 12:** Minimum Demand Power Factor for Bus 14 and 9: Detail.

Bus	Details	P (kW)	Q (kW)	PF
MV bus 14	Bus 14_1	393,84	363,6	0,73
	Load 14	372	279	0,8
	MV Bus 14	21,84	84,6	0,23
MV bus 9	MV bus 9	110,2	0,69	1

In both scenarios, it demonstrates that there is a great deterioration of the power factor in bus 14 because this bus is a transfer bus as shown in Fig. 18. Tables 11 and 12 show the results of the power flows in bus 14, where it can be seen that the measurement of flows in this bus contains the flows of load 14 and the transfer flow to line 14, which is transporting a reactive power higher than the active power, as shown in Tables 11 and 12 for each scenario. A similar situation occurs in bus 5 as it only operates as a transfer bus, connecting line 2, line 3 and line 5. This bus is also transporting a reactive power higher than the active power. BESS #2 alone is operating at 0.8 lagging.

The comparison of power factors between the two scenarios shows some results that are particularly important. For example, in conditions of minimum demand, the power factor is much better in most of the buses than the power factor in the maximum demand scenario. The relation of decrease in the reactive power of the maximum demand scenario to the minimum demand scenario is the same relation of decrease for reactive power. With this analysis it might seem that the power factor seen by the grid should be the same. However, the power factor in maximum demand is 0,73 while in minimum demand, it is 0,77.

This remarkable difference between the power factors in both scenarios can also be seen in buses 5 and 6. This phenomenon is due to the loss of active power contribution in the solar photovoltaic generations, which do not occur in the scenario of minimum demand that takes place at night when there is no solar radiation. This analysis shows the conflict that can exist in the variable power factor when it comes to compensating a power electrical system only with the contribution of active power. With the injection of only active power, the power factor seen by the grid deteriorates, which leads to the need to compensate a system with more criteria and where joint compensation of active and reactive power is considered.

The power factor in bus 7 in the scenario of minimum demand shown in Fig. 19 is at a lower value with respect to the power factor in the same bus in maximum demand, this is due to the contribution of reactive power of the Diesel generator depending on the power flows.

### 5.6 THD per bus

**Fig. 20:**
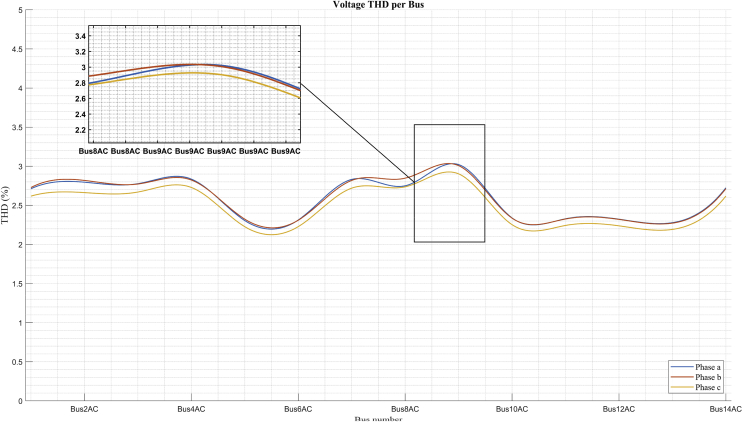
THD per bus in maximum demand.

**Fig. 21:**
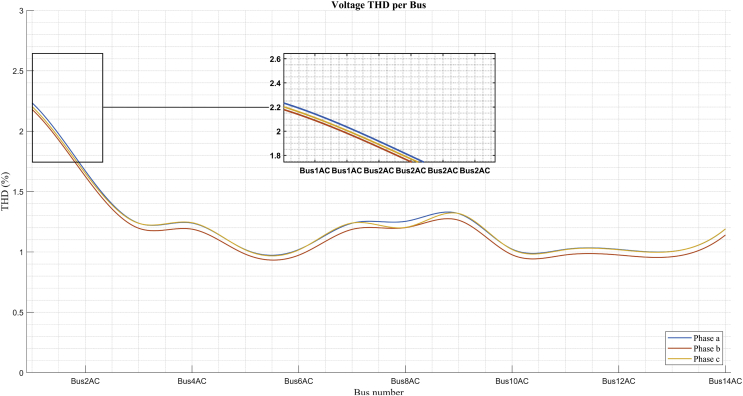
THD per bus in minimum demand.

This section presents the analysis of the deformation of the sine wave voltage. This is one of the problems that affects the quality of electric power especially in distribution systems. Figs. 20 and 21 show the Harmonic Distortion Index (THD) of the voltage for each of the buses of the system and in each of the phases, in the scenarios of maximum demand and minimum demand, respectively. It can be seen in both figures that the harmonic distortion does not exceed 3,5 % for any of the calculated scenarios. However, these values are a base case for future studies with insertion of non-linear loads and for optimization problems that involve the improvement of THD and the compensation of the reactive by means of equipment based on power electronics. The THD in each bus is calculated as shown in Eq. (21) [61, 62, 63].

(21) $$THD\%=\frac{\sqrt{{\sum_{i=2}^{H}\left(V_{i,h}\right)}^{2}}}{V_{i,1}}*100$$

where:

$V_{i,h}$ is the voltage component corresponding to the harmonic *h* at the node i;
$V_{i},1$ is the fundamental voltage component (1^st^ harmonic) at the node i;
H, is the maximum harmonic order to be considered in the calculation.

## 6 Future work and research areas

As in [4], international organizations such as Power Systems Engineering Research Center (PSERC), Consortium for Electric Reliability Technology Solutions (CERTS), Consortium for Electric Reliability Technology Solutions (NEDO), Transmission and Distribution Research Center (T&D) among others, have presented research projects in Smart MGs to the scientific community. All these entities have the aim of guaranteeing the speed of the grid and the quality of the energy, integration of resources and distributed generation, generation of active Distribution Grids, emissions reduction, decrease of construction and investment times, and reduction of losses in T&D.

In addition, the nature of modern power grids has gone from being dominated by a few generation units to a grid dominated by many smaller generating units, together bringing a growing number of renewable generation sources such as wind turbines, photovoltaic systems, and energy storage systems. This has led to great research efforts in power grids and smart MGs to mitigate new challenges such as: low and erratic consumption, non-existent decoupling of P – f - |V|; the loss of coordination of MG protection; voltage unbalance conditions; increased voltage; the connection/disconnection of DG sources; high failure rates, among others.

As it has been commented in the introduction, some of the interfacing converters are in an open control loop, i.e. no control over the index modulation nor over the phase angle has been implemented. In a previous study, this technique was successfully used to establish a certain operation point [64]. This is a pending issue for the benchmark [65]. There are other tests for the benchmark [66, 67].

This study offers the possibility of developing in research areas that must be consider:

• Grid-islanded mode [44, 68], synchronization modes to the Main Grid [24, 69], tests over the MG controllability in the islanded configuration [70], MG modelling, and analysis [10, 47, 71, 72, 73], tests over reactive compensation as a solution for single phase non-linear loads like electric arc furnaces using Static Var Compensation (SVC) [29], tests over power quality issues. Explore the possibility of using Distribution STACOM, Green Plug Filter Compensators (GPFC) or Modulated Power Filter Compensators (MPFC) [2, 74], tests over optimization and sizing techniques [75, 76, 77], control of power electronic converters [DC MG Control (boost-buck)] and hierarchical control structure tests [1, 21, 30, 38, 78, 79, 80, 81, 82, 83, 84].

Another critical problem in MGs is the growth in the number of failures in their components, especially the failures that comprise the infrastructure of control and automation, communications, generation, and distribution of energy.

In the future, fault modeling and analysis may be performed which not only allows us to understand the various failure modes (their causes and effects) but also to study optimal methodologies for taking timely detection, diagnosis and localization actions.

The objective is to develop and sustain the development of real-time automated tool research that allows mitigation and failure relief [85], the development of fault-tolerant control strategies [78, 86, 87, 88, 89, 90, 91, 92, 93, 94, 95, 96, 97, 98, 99, 100, 101, 102, 103], intelligent and adaptive protections in AC and DC [6, 104], among others. The support of these projects such as those described in [85, 86, 93], confirms and shows dynamic behaviors that benefit engineers that work in the area of fault diagnosis as well as those that develop fault tolerance methodologies for control, which in turn helps to improve the resilience and reliability of the MGs. Some development in predictive [105, 106, 107, 108, 109] and hierarchical control techniques [20, 38, 40, 80, 82, 110, 111, 112, 113] can be explored in this approach, among others. The support of these projects such as those described in [85, 86, 93], shows dynamic behaviors that benefit engineers that work in the area of fault diagnosis as well as those that develop fault tolerance methodologies for control. This in turn helps to improve the resilience and reliability of the MGs. Some developments in predictive [109, 110, 111, 112, 113] and hierarchical control techniques [20, 38, 40, 80, 82, 105, 106, 107, 108] can be explored in this approach.

In addition, the base case obtained in this research with maximum and minimum demand scenarios allows future studies in many areas of great current interest, associated with the efficiency, reliability and quality of the electric power. The proposed system allows the analysis of power dispatch in the event of grid contingencies or unbalances between load and generation. The impact of the insertion of the electric car in the demand studies of both scenarios can be analyzed.

This model may be the base for future studies such as optimal dimension and location of reactive power compensating devices. It is also possible to analyze the reliability indicators and grid expansion proposals for future load growth. In addition, dynamic analysis of the stability of the frequency could be carried out for disturbances, faults and unbalances between generation and load, especially considering the loss of inertia in the system with isolated mode.

## 7 Conclusions

In this paper, a typical AC/DC HMG benchmark based on the IEEE 14 node test feeder is presented. The selection of renewable energy sources have considered the availability of photovoltaic energy resources instead of wind energy resources. This AC/DC HMG benchmark includes a one-line diagram as well as essential data for the 13,8-kV primary system and 0,22-kV secondary system. The proposed study is subjected to two scenarios: minimum and maximum power demands. Both power flow results are published for each scenario. All buses have their measurement module. Simulation of measurements related with power quality indexes as current and voltage total harmonic distortion (THDV and THDI) are calculated, including capability to compute power line losses and power factor. The results obtained in the analysis of the two scenarios calculated in this proposed model have been discussed individually, allowing a wide understanding of the problem of each of the variables involved in the analysis of efficiency and quality of the power. The conflict that exists in some of the variables was analyzed, especially in the power factor, due to the high penetration of photovoltaic solar generation, which can cause a deterioration of the power factor seen by the commercial entities that provide the electric service in the mode connected to the grid.

In the future, MGs are an attractive solution for the integration of DG units in the Smart Grid and may help lessen a dependence on fossil fuels and increase the efficiency of the electrical grid. However, the challenges are still present due to the following factors: the rapid dynamics and short response time of DG or distributed energy resources, the inherent unbalanced nature of the MG, low energy storage capacity and lack of inertia, a high number and diversity of micro sources used, electronic device power converters and other circuits/devices, a high degree of parametric uncertainties, modelling, and high failure rates.

This benchmark study opens the possibility to investigate transient stability, test control strategies and hierarchical control structure, explores isolated scenarios, simulates the dynamics of the diesel generator, compensates voltage profiles, among others. The investigation allows to deepen the scenarios and topologies of the interconnected AC/DC HMGs and allows dynamic studies for other AC/DC HMG configurations, even though it depends on a large extent on the application and the integrated environment (market conditions, regulatory conditions, feasibility for the integration of DG units, among others). Finally, this study constitutes a base to develop research in the framework of MGs, its potential applications, as well as improve reliability, efficiency and device costs.

## Declarations

### Author contribution statement

Leony Ortiz, Rogelio Orizondo & Alexander Águila: Conceived and designed the experiments; Performed the experiments; Analyzed and interpreted the data; Contributed reagents, materials, analysis tools or data; Wrote the paper.

Jorge W. González, Gabriel J. López & Idi Isaac: Conceived and designed the experiments; Analyzed and interpreted the data; Contributed reagents, materials, analysis tools or data; Wrote the paper.

### Funding statement

This research did not receive any specific grant from funding agencies in the public, commercial, or not-for-profit sectors.

### Competing interest statement

The authors declare no conflict of interest.

### Additional information

No additional information is available for this paper.
